# Automated assessment reveals that the extinction risk of reptiles is widely underestimated across space and phylogeny

**DOI:** 10.1371/journal.pbio.3001544

**Published:** 2022-05-26

**Authors:** Gabriel Henrique de Oliveira Caetano, David G. Chapple, Richard Grenyer, Tal Raz, Jonathan Rosenblatt, Reid Tingley, Monika Böhm, Shai Meiri, Uri Roll

**Affiliations:** 1 Jacob Blaustein Center for Scientific Cooperation, The Jacob Blaustein Institutes for Desert Research, Ben-Gurion University of the Negev, Midreshet Ben-Gurion, Israel; 2 Mitrani Department of Desert Ecology, The Jacob Blaustein Institutes for Desert Research, Ben-Gurion University of the Negev, Midreshet Ben-Gurion, Israel; 3 School of Biological Sciences, Monash University, Clayton, Victoria, Australia; 4 School of Geography and the Environment, University of Oxford, Oxford, United Kingdom; 5 School of Zoology and Steinhardt Museum of Natural History, Tel Aviv University, Tel Aviv, Israel; 6 Ben-Gurion University of the Negev, Beer Shiba, Israel; 7 Institute of Zoology, Zoological Society of London, London, United Kingdom; 8 Global Center for Species Survival, Indianapolis Zoological Society, Indianapolis, Indiana, United States of America; Estacion Biologica de Doñana CSIC, SPAIN

## Abstract

The Red List of Threatened Species, published by the International Union for Conservation of Nature (IUCN), is a crucial tool for conservation decision-making. However, despite substantial effort, numerous species remain unassessed or have insufficient data available to be assigned a Red List extinction risk category. Moreover, the Red Listing process is subject to various sources of uncertainty and bias. The development of robust automated assessment methods could serve as an efficient and highly useful tool to accelerate the assessment process and offer provisional assessments. Here, we aimed to (1) present a machine learning–based automated extinction risk assessment method that can be used on less known species; (2) offer provisional assessments for all reptiles—the only major tetrapod group without a comprehensive Red List assessment; and **(**3) evaluate potential effects of human decision biases on the outcome of assessments. We use the method presented here to assess 4,369 reptile species that are currently unassessed or classified as Data Deficient by the IUCN. The models used in our predictions were 90% accurate in classifying species as threatened/nonthreatened, and 84% accurate in predicting specific extinction risk categories. Unassessed and Data Deficient reptiles were considerably more likely to be threatened than assessed species, adding to mounting evidence that these species warrant more conservation attention. The overall proportion of threatened species greatly increased when we included our provisional assessments. Assessor identities strongly affected prediction outcomes, suggesting that assessor effects need to be carefully considered in extinction risk assessments. Regions and taxa we identified as likely to be more threatened should be given increased attention in new assessments and conservation planning. Lastly, the method we present here can be easily implemented to help bridge the assessment gap for other less known taxa.

## Abstract 2

The Red List of Threatened Species, published by the IUCN, is a crucial tool for conservation decision making, but is subject to various sources of uncertainty and bias. Modelling the threat status of all global reptiles identifies increased threat to many groups of reptiles across many regions of the world, beyond those currently recognized; moreover, it highlights the effects of the IUCN assessment procedure on eventual threat categories.

## Introduction

The International Union for Conservation of Nature’s (IUCN) Red List of Threatened Species [1] is the most comprehensive assessment of the extinction risk of species worldwide [2]. Since its inception in 1964, the Red List has been instrumental in “generating scientific knowledge, raising awareness among stakeholders, designating priority conservation sites, allocating funding and resources, influencing development of legislation and policy, and guiding targeted conservation action” [3]. For example, the 2004 completion of IUCN’s Global Amphibian Assessment reported their dire global state [4] and led to the creation of organizations dedicated to amphibian conservation and to increased funding for research and conservation policy focused on amphibians [3]. Additionally, the IUCN’s Red List forms a basis for the designation of priority areas for conservation, such as Key Biodiversity Areas [5]. For example, the Alliance for Zero Extinction [6] works directly with decision-makers to establish protected areas for threatened species represented by a single population, using Red List data.

The Red List assigns evaluated species to categories based on their distribution, population trends, and specific threats [7]. The categories Least Concern (LC) and Near Threatened (NT) are deemed not threatened, while Vulnerable (VU), Endangered (EN), and Critically Endangered (CR) species are deemed threatened. Other species are assessed as Extinct in the Wild (EW), Extinct (EX), or Data Deficient (DD). DD category is assigned to species for which information is insufficient to assign them any of the above categories. Still, most of global biodiversity remains Not Evaluated (NE) by the Red List. This is predominantly due to the laborious nature of Red List assessments, which are based on voluntary expert participation, usually through multiparticipant in-person meetings [7]. Importantly, NE and DD species are generally not prioritized for conservation decision-making, although Red List guidelines specifically state that they “should not be treated as if they were not threatened” [7]. Even though DD species have been shown to be comparable to CR ones with respect to their levels of overlap with human impact [8]. These assessment gaps [9,10] led to the use of several automated methods to provisionally assess species [11,12]. These methods employ algorithms including phylogenetic regression models [13–15], structural equation models [16], random forests [17,18], deep learning [19,20], Bayesian networks [21,22], and even linguistic analysis of Wikipedia pages [23]. Most previous attempts (e.g., [13,17,18]) employed a binary classification of threatened (categories CR, EN, and VU) versus nonthreatened (NT and LC). Few studies attempted to predict specific categories (e.g., [19,20,24]), which are more useful to decision makers as they enable prioritizing among threatened species. A more comprehensive review of these methods [25] also calls for attention to obstacles for their implementation in the assessment process. This review argues that a major obstacle for their implementation is the lack of communication between conservation researchers developing such methods and IUCN personnel [25].

A challenge that remains unaddressed in automated assessment is human decision bias. Biases are introduced by ambiguities in the interpretation of IUCN guidelines by assessors and reviewers, heterogeneity in assessor expertise levels, and personal agendas [26]. The IUCN tries to decrease reliance on subjective expert opinions [2], even employing automated assistance for generating and verifying assessments [12]. However, expert input (and guidance from the IUCN personnel who lead each workshop) remains an important part of the assessment process. Automated methods that ignore such biases in their training data risk reproducing or even amplifying them in their predictions [27].

Reptiles remain the only tetrapod group without comprehensive IUCN assessment. As of July 2021, approximately 28% of 11,570 reptile species remain unassessed and approximately 14% of those assessed have been classified as DD [1] Moreover, many of the reptile assessments are more than 10 years old rendering them outdated as per IUCN guidelines [1]. This assessment gap is not random. Smaller species, with narrow distributions, located in the tropics, are less likely to have been assessed [9]. Bland and Böhm [28], and Miles [19], automatically assessed some reptile species. Their models predicted approximately 20% of NE and DD species are threatened, a similar proportion to those assessed as such (excluding DD). However, in both studies, models were trained and validated using a small set of species with a wealth of morphological, ecological, and life history data (which are rare for DD species). Such exercises might provide important information on the mechanisms underlying extinction risk. However, these data-hungry methods are greatly limited in their utility because such data are unavailable for the vast majority of DD and NE species (e.g., DD and newly described reptiles, most invertebrate taxa). Ultimately, we need methods that will enable precise automated extinction risk assessments of species, which acknowledge different biases and data gaps.

Here, we use robust machine learning to automatically predict IUCN extinction risk categories to all reptile species globally, to (1) present a new automated assessment framework and (2) provisionally fill the reptile assessment gap. Our methods rely only on readily available data (mostly geographic ranges, phylogenetic structure, and body mass) and estimate potential effects of assessor or reviewer identities. We use these methods to assign provisional extinction risk categories to 4,369 reptile species, of which 3,286 are currently unassessed and 1,083 are currently classified as DD. We further explore global trends in extinction risk across all reptiles and highlight the effects of our new provisional categories on overall patterns in this class. Lastly, we highlight potential sources of biases and incongruences in the assessment process.

## Results

### General model results

We implemented a novel automated assessment method, using the XGBoost algorithm [29], and provided provisional assessment to 4,369 reptile species that were previously NE or assessed as DD (S1 Data). Of these 4,369 species, we assessed 1,161 (27%) as threatened (244 as CR, 467 as EN, and 450 as VU), and 3,208 as non-threatened (3,021 as LC and 187 as NT). This is compared to 21% threatened species in the assessed/training dataset (1,375 of 6,520, χ^2^: 26.947, *p*-value: <0.001).

The model we used to predict extinction risk for DD and NE species included spatial and phylogenetic autocorrelation and excluded assessor/reviewer effects, achieved 90% validated accuracy for the binary threatened/nonthreatened classification, and 84% accuracy for predicting specific categories (AUC - Area Under Curve: 0.83, Tables 1 and 2). The complete model, including spatial and phylogenetic autocorrelation, and assessor/reviewer effects, achieved similar results, as did the model excluding spatial and phylogenetic autocorrelation but retaining assessor/reviewer effects (Table 1). The model excluding both autocorrelations and assessor/reviewer effects, and the models including either spatial or phylogenetic autocorrelation, were less accurate (Table 1). However, the model obtained the highest accuracies when excluding threatened species classified under criteria other than B from the training dataset (Table 1; details below). We predicted extinction risk categories for DD and NE species using the model that excluded assessor/reviewer effects but retained spatial and phylogenetic data, since we cannot know the identity of assessors who will evaluate currently unassessed species. For analyses regarding potential assessor/reviewer effects, we used the complete model. Detailed accuracy metrics are presented in Table 2. The lowest accuracy across models was in separating the NT and LC categories (Table 2).

**Table 1:** Comparison of accuracy metrics of 8 automated assessment models for classifying reptile species into IUCN extinction risk categories.

Model	Task	Species sampling	Predictors	Accuracy	AUC
Complete	Binary	all	Environmental + body mass + PEM + MEM + assessor/reviewer effects	0.904	0.833
	Specific	all	Environmental + body mass + PEM + MEM + assessor/reviewer effects	0.852	0.812
Environment and body mass	Binary	all	Environmental + body mass	0.877	0.784
	Specific	all	Environmental + body mass	0.821	0.777
Assessor/reviewer effects	Binary	all	Environmental + body mass + assessor/reviewer effects	0.890	0.805
	Specific	all	Environmental + body mass + assessor/reviewer effects	0.835	0.802
Spatial	Binary	all	Environmental + body mass + MEM	0.889	0.807
	Specific	all	Environmental + body mass + MEM	0.825	0.791
Phylogenetic	Binary	all	Environmental + body mass + PEM	0.884	0.800
	Specific	all	Environmental + body mass + PEM	0.826	0.781
**Spatial-phylogenetic (used for prediction)**	**Binary**	**all**	**Environmental + body mass + PEM + MEM**	**0.900**	**0.828**
	**Specific**	**all**	**Environmental + body mass + PEM + MEM**	**0.837**	**0.801**
Complete—Criterion B	Binary	Criterion B + NT, LC	Environmental + body mass + PEM + MEM + assessor/reviewer effects	0.926	0.838
	Specific	Criterion B + NT, LC	Environmental + body mass + PEM + MEM + assessor/reviewer effects	0.875	0.803
Spatial-phylogenetic—Criterion B	Binary	Criterion B + NT, LC	Environmental + body mass + PEM + MEM	0.915	0.800
	Specific	Criterion B + NT, LC	Environmental + body mass + PEM + MEM	0.858	0.782

The “complete” model includes environmental predictors, body mass, spatial and phylogenetic autocorrelations, and assessor/reviewer effects. The model used to predict extinction risk for DD and NE species—“Spatial-phylogenetic” model—includes environmental predictors, body mass, and spatial and phylogenetic autocorrelations but excludes assessor/reviewer effects, as this information is not available for unassessed species. The species sampling column indicates which species were used in the training of each model, in regard to their extinction risk category and criteria used by IUCN on their assessment. The “Binary” task represents the separation of threatened (CR, EN, and VU) from nonthreatened categories (NT and LC). The “Specific” task represents classification into IUCN extinction risk categories. MEM and PEM represent spatial and phylogenetic autocorrelations, respectively. More detailed metrics are presented in Table 2.

AUC, Area Under Curve; CR, Critically Endangered; DD, Data Deficient; EN, Endangered; IUCN, International Union for Conservation of Nature; LC, Least Concern; MEM, Moran’s Eigenvector Maps; NE, Not Evaluated; NT, Near Threatened; PEM, Phylogenetic Eigenvector Maps; VU, Vulnerable.

**Table 2:** Accuracy metrics of automated assessment models classifying reptile species into IUCN extinction risk categories, under 2 different approaches: (1) complete model, accounting for spatial and phylogenetic autocorrelation and assessor/reviewer effects; (2) accounting for spatial and phylogenetic autocorrelation (this was the model used for predictions).

	Binary	CR	EN	VU	NT	LC
**Complete**						
Sensitivity	0.955	0.773	0.699	0.532	0.278	0.964
Specificity	0.711	0.997	0.976	0.977	0.983	0.691
AUC	0.833	0.885	0.837	0.755	0.631	0.828
Precision	0.925	0.927	0.731	0.649	0.556	0.890
Recall	0.955	0.773	0.699	0.532	0.278	0.964
F1	0.940	0.843	0.715	0.585	0.370	0.925
**Spatial-phylogenetic (used for predictions)**						
Sensitivity	0.952	0.621	0.726	0.278	0.532	0.950
Specificity	0.703	0.995	0.969	0.976	0.979	0.683
AUC	0.828	0.808	0.847	0.627	0.756	0.816
Precision	0.923	0.872	0.689	0.463	0.667	0.886
Recall	0.952	0.621	0.726	0.278	0.532	0.950
F1	0.938	0.726	0.707	0.347	0.592	0.917

“Binary” represents the separation of threatened (CR, EN, and VU) from nonthreatened categories (NT and LC). Remaining columns represent the predictive accuracy for assigning species to the 5 extinction risk categories: CR, Critically Endangered; EN, Endangered; LC, Least Concern; NT, Near Threatened; VU, Vulnerable. See S1 Table for remaining models.

Across different classification tasks and extent of occurrence classes, the average ranking of the importance of feature classes in the complete model was predominantly due to (1) spatial autocorrelation; (2) assessor effects; (3) phylogenetic autocorrelation; (4) climate; and (5) human encroachment. In the model excluding assessor/reviewer effects, the ranking was: (1) spatial autocorrelation; (2) phylogenetic autocorrelation; (3) climate; (4) human encroachment; and (5) insularity (for full details on feature importance across models, see S1 Fig and S2 Table; for a list of variables in each category, see S1 Data). The hyperparameter configuration for the model chosen for predictions is summarized in S3 Table. The features selected for each combination of range size (calculated as extent of occurrence) class and classification task are provided in S1 Data. The contribution of each feature class to predictive performance for each combination of range size class and classification task is presented in S1 Fig.

Criterion B for IUCN extinction risk assessments—which is predominantly based on species range sizes [7]—is the most widely used criterion for assigning a threatened status in reptile assessments (74% of species assessed under any criteria). The model only trained on species assessed as threatened based on criteria B, as well as NT and LC species, was more accurate for both binary (93%, AUC: 0.84, Table 1) and specific categorizations (87%, AUC: 0.80, Table 1). Further, excluding assessor/reviewer effects resulted in similar accuracy (binary classification: 92% accuracy, 0.80 AUC; specific classification: 86% accuracy, 0.78 AUC; Table 1). Despite their higher accuracy, these models tended to misclassify non-criterion B–threatened species, assigning them to lower extinction risk categories than observed (S4 Table). This is probably because species are only classified under non-B criteria if such criteria assign them to a similar, or higher, extinction risk category. Thus, we proceeded with models trained on all species for the remaining analyses. Our model correctly classified 93.8% of previously assessed species (6,112 of 6,520 species). The 6.2% misclassified species (408 of 6,520 species) were nearly twice as likely to be assigned to nonthreatened categories than to shift in the opposite direction and generally to shift to less threatened specific categories (S2 Fig). This was consistent in most biogeographical realms, except in the Nearctic and Neotropical realms, in which the numbers were similar for the binary classification (S2 Fig).

### Comparison with previous methods

We compared our method to similar past endeavors. Our simplest model (“Environment and body mass”; Table 1) obtained higher accuracy (88%) than methods based on Random Forest (85%) and Neural Networks (79%), using the same predictors (S5 Table). The extreme class imbalance in the dataset greatly hindered both methods, especially Neural Networks (S5 Table), despite the use of supersampling to account for uneven class distributions. In fact, Neural Networks are known to be sensitive to such imbalances [30], while XGBoost is considered more robust to them [29]. While previous methods have incorporated similar predictors to ours, and have separately incorporated features such as tolerating missing values, identifying specific IUCN categories, and accounting for spatial and phylogenetic autocorrelation, none did so in combination, as our method did (S6 Table). Our method is also the first to account for assessor bias (as an exploratory tool, not for prediction; S6 Table).

### Predictions for data deficient and not evaluated species

**Fig 1:**
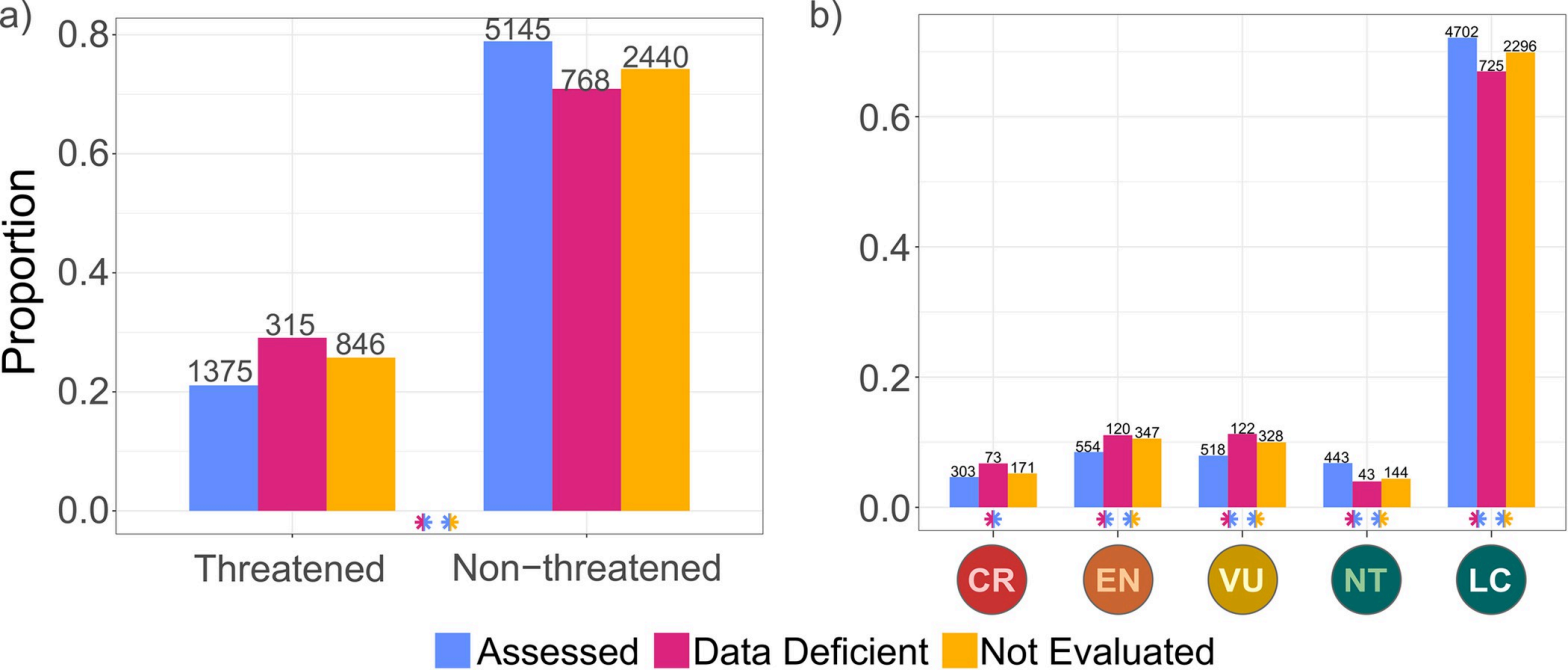
Proportion of reptile species assigned to extinction risk categories by IUCN manual assessment (assessed) and by an automated assessment model (Data Deficient and Not Evaluated). (A) Grouping categories into threatened and nonthreatened and (B) specific extinction risk categories: CR, Critically Endangered; EN, Endangered; LC, Least Concern; NT, Near Threatened; VU, Vulnerable. Number of species in each category is indicated above each bar. Significant differences in a Pearson’s χ^2^ test are indicated by asterisks, colored according to which proportions are being compared (S7 Table). The data underlying this figure can be found in S2 Data.

DD and NE species were significantly more likely to be assigned threatened categories than assessed species (DD: 29%, NE: 26%, assessed non-DD: 21% threatened; Fig 1A, S7 Table). DD species were more likely than assessed species to be predicted as VU, EN, or CR, and less likely to be predicted as NT or LC. NE species were more likely than assessed species to be VU, and EN, and less likely to be predicted as NT or LC (Fig 1B, S7 and S8 Tables).

### Phylogenetic and spatial patterns

**Fig 2:**
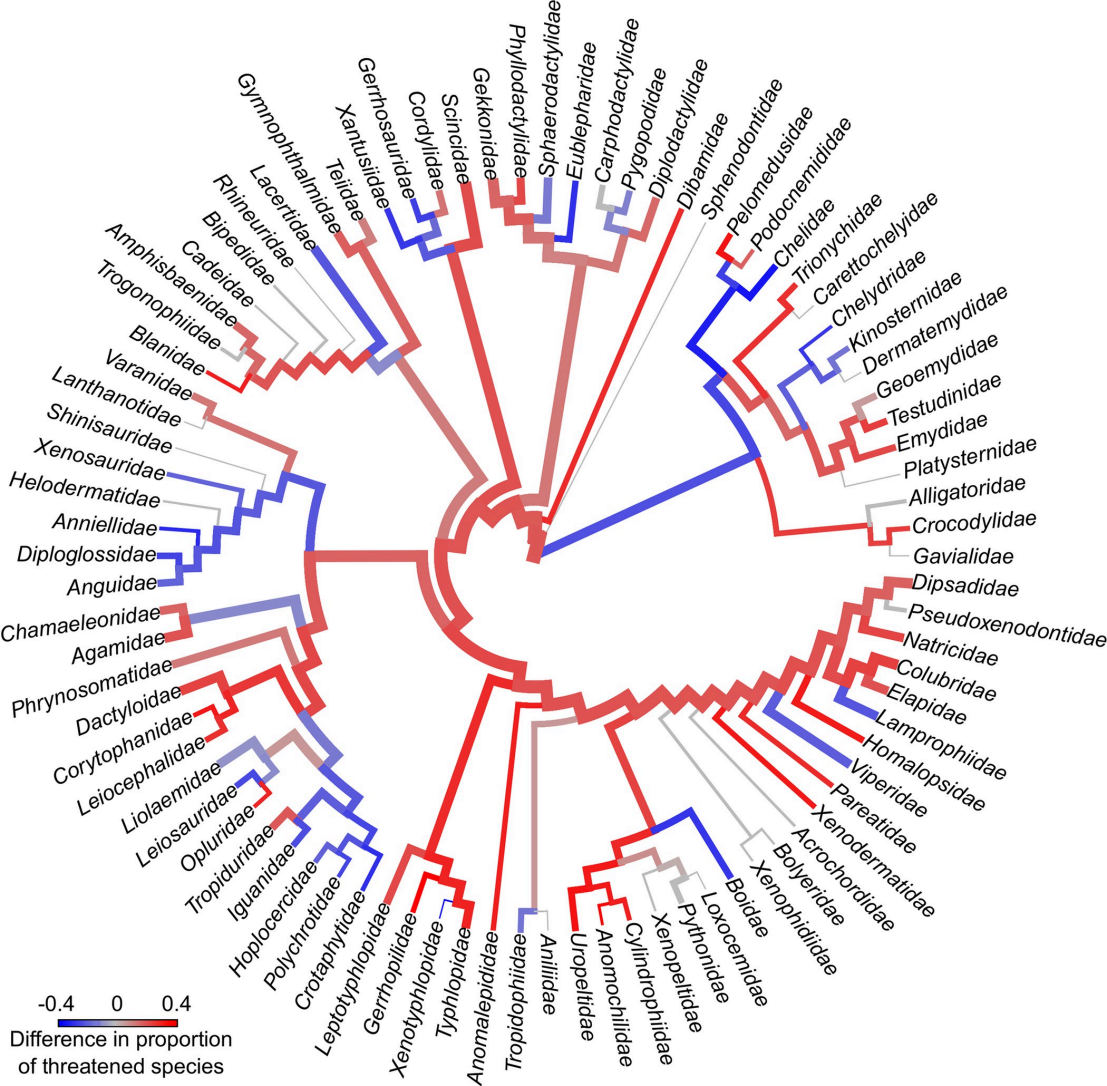
Differences in the percentage of threatened species in reptile families before and after the addition of extinction risk estimates for DD and NE species, obtained from an automated assessment method. Colors in internal nodes represent the difference in percentages for all descendant tips. Trees by Tonini and colleagues [31] (Squamata) and Colston and colleagues [32] (Archelosauria). The shift between red and blue is proportional to the (symmetric log scale) increase/decrease in extinction risk per branch when using our assessments. Branch widths are proportional to log species richness in each clade. Proportion of threatened species for each family, before and after inclusion of automated assessments are detailed in S9 Table. The data underlying this figure can be found in S2 Data. DD, Data Deficient; NE, Not Evaluated.

**Fig 3:**
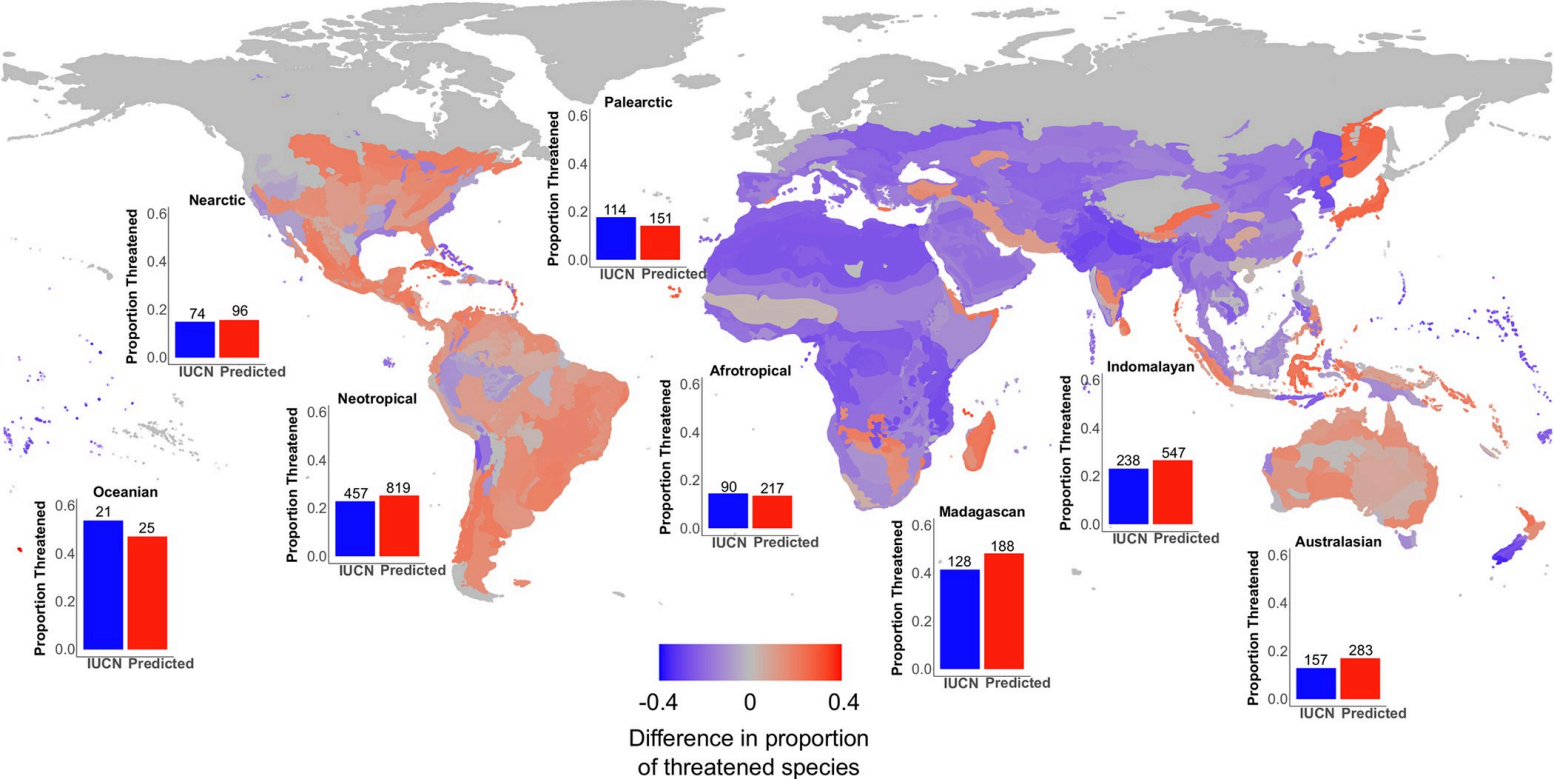
Global spatial changes in the percentage of threatened reptile species resulting from our automated assessments. The spatial data are grouped by WWF terrestrial ecoregions. The shift between red and blue is proportional to the (symmetric log scale) increase/decrease in extinction risk per ecoregion when using our assessments. Bar plots indicate proportion of species in threatened categories for each biogeographical realm, before and after the inclusion of automated assessments. The data underlying this figure can be found in S2 Data. IUCN, International Union for Conservation of Nature; WWF, World Wide Fund for Nature.

The proportion of threatened species increased overall for Squamata and Crocodylia, but decreased for Testudines (Fig 2, S9 Table), especially in the turtle families Chelidae, Chelydridae, and Kinosternidae. Anguimorph lizards (except Varanidae) proportion of threatened species decreased following our predictions. The 3 largest lizard clades—Iguania, Scincomorpha, and Gekkota—(as well as Lacertoidea except Lacertidae) showed increased threat, as did the largest snake clades (Colubridae, Dipsadinae, Elapidae) and Serpentes as a whole (Fig 2, S9 Table). Including predictions for DD and NE species, the proportions of threatened species increased in ecoregions across most of South and North America, Australia, and Madagascar (Fig 3, S10 Table).

### Effect of assessor/reviewer identities on predictions

**Fig 4:**
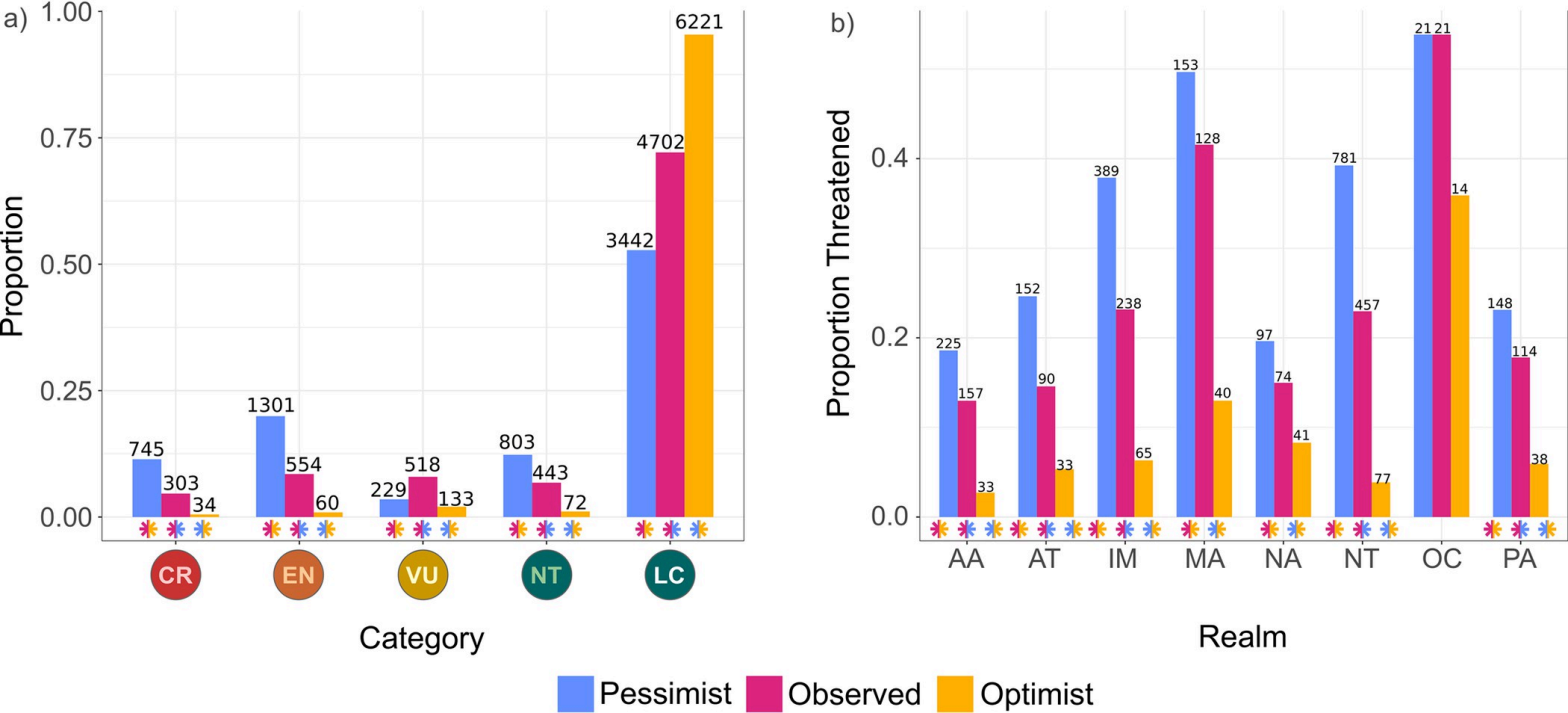
Proportion of threatened reptile species under different assessor bias scenarios. Analysis includes only species that have IUCN assessments (6,520 species). (a) Proportion of reptile species assigned to each extinction risk category for the actual IUCN assessments (Observed); proportion expected if the most optimistic group of assessors assessed every species (Optimistic); proportion expected if the most pessimistic group assessed every species (Pessimistic). (b) Proportion of threatened species in each biogeographical realm for Observed, Optimistic, and Pessimistic assessments. Significant differences in a Pearson’s χ^2^ test are indicated by asterisks, colored according to which proportions are being compared (S11 Table). The data underlying this figure can be found in S2 Data. AA, Australasian; AT, Afrotropical; CR, Critically Endangered; EN, Endangered; IM, Indomalayan; LC, Least Concern; MA, Madagascan; NA, Nearctic; NT, Near Threatened; NT, Neotropical; OC, Oceanian; PA, Palearctic; VU, Vulnerable.

We permuted the identity of assessors and reviewers until we identified the group of assessors and reviewers that would assign each species to the least threatened category possible, while maintaining the other predictors’ values (optimistic scenario) and to the most threatened category possible (pessimistic scenario). Proportions of species predicted as threatened increased from optimistic to observed to pessimistic scenarios for all categories (Fig 4A, S11 Table) and across most biogeographical realms. In the Nearctic and Madagascar, the observed and pessimistic scenarios were similar, and in Oceania no differences were detected (Fig 4B, S12 Table). Species that changed category between the observed assessments and the optimistic scenario moved overwhelmingly to a single category (LC), while in the pessimistic scenario, species showed a more diverse distribution of new categories (S3 Fig).

## Discussion

Our model assigned IUCN extinction risk categories to the 40% of the world’s reptiles that currently lack published assessments or are classified as DD. Our novel modeling approach enabled classifying specific extinction risk categories with high accuracy using only readily available data (ranges and body sizes). Our methods also gained better accuracy than previously explored methods (S5 Table). We predicted that the prevalence of threatened reptile species is significantly higher than currently depicted by IUCN assessments. This pattern is widespread across space and phylogeny. Our results show that, while high prediction accuracy can be achieved without explicitly accounting for assessor/reviewer identities, the identity of assessor/reviewers greatly affects predictions.

### General model results

The classification accuracy of more extreme categories (CR, EN, and LC) was higher than categories straddling the threatened/nonthreatened threshold (VU and NT; S1 Table). This likely reflects ambiguities inherent to the assessment of borderline cases, while extreme cases are easier to identify. This is compounded in the category it proved hardest to predict (NT), as there are no distinct quantitative thresholds for NT as there are for threatened categories (although guidance is given by the IUCN on how NT should be assessed [7]). Such thresholds are a primary factor for assigning criterion B extinction risk designations (and for our modeling). Misclassifications of assessed species tended toward less threatened categories (S2 Fig) indicating that our predictions of unassessed species may actually be more optimistic than the true state of extinction risk for reptiles.

Machine learning methods, such as XGBoost, are geared primarily toward prediction not inference [33]. Any ecological interpretation of feature importance should thus be taken with caution. The greater importance of spatial and phylogenetic eigenvectors in our classification tasks (S1 Fig, S2 Table) is most likely due to the greater number of features included in these categories. Nevertheless, this shows that extinction risk has highly predictable spatial and phylogenetic patterns, i.e., that some regions and some taxa are more prone to extinction than others. This can be used to approximate the conservation status of less studied taxa, for which no other information is available. The climatic and human encroachment variables obtained high importance scores. A previous meta-analysis found widespread negative effects of human land modification on reptile abundance but no effect of climate [34]. This discrepancy could be due to climate acting as proxy for other highly spatially autocorrelated factors. Insularity was also important in many of the classification tasks in agreement with previous studies that identified it as a major contributor to extinction vulnerability in reptiles [35]. Range size, another major correlate of extinction risk, did not rank high in our models, likely due to it already being used as an a priori criterion to separate species before training models. Future studies should expand on the mechanisms underlying the spatial and phylogenetic patterns in extinction risk identified in this study.

Nine species classified as CR by IUCN were considered LC by our model. Some of these have fragmented ranges (*Spondylurus lineolatus*, *Liolaemus azarai*, and *Emoia slevini*), which might have caused our model to underestimate their extinction risk. Our models used extent of occurrence as a proxy of range size, which can greatly differ from area of occupancy in species with fragmented ranges. Thus, species evaluated under area of occupancy criteria might be harder to capture in our model. Small and fragmented ranges can also be more unstable, which might result in discrepancies between the datasets used to train the model. GARD range data represents historical ranges, including parts of the range from which populations may have been extirpated. This might cause some of the discrepancies observed. For example, the GARD database includes range fragments of *S*. *lineolatus* that are classified as possibly extinct in the IUCN database.

Other species classified as less threatened by the model suffer from threats such as invasive species (*Liolaemus paulinae* and *Cyrtodactylus jarakensis*), quarrying (*Homonota taragui* and *Cyrtodactylus guakanthanensis*), tourism (*Calamaria ingeri*), and fires (*Bellatorias obiri*), which are not accounted for in our modeling. Although some of the human encroachment features included might act as proxies for such threats, some local stressors will escape this approximation.

Four species (*Tropidophis xanthogaster*, *Cubatyphlops perimychus*, *Celestus marcanoi*, and *Chioninia spinalis*) were classified as LC by IUCN, but as CR by our model. All are small ranged species located in protected areas. Protected area effects, and local population dynamics may not have been captured by our model in rare cases, leading to occasional overestimation of threat. Alternatively, actual assessments may have been inconsistent with most of the Red List. These are poorly known species, their IUCN assessments read: “*while threats have been identified*, *these are presently localized*” (*T*. *xanthogaster)*; *“the limited information available indicates that it is able to adapt at least to certain forms of disturbance”* (*C*. *perimychus)*; *“there is no information about its population… Further research into its distribution*, *abundance*, *and population trends should be carried out to have more knowledge about how the threats are impacting the species”* (*C*. *marcanoi)*. This lack of information opens room for the introduction of biases, such as overly optimistic assessors overlooking important threats. All 4 species classified as LC by IUCN and CR by our model have extremely restricted ranges and are endemic to islands with high proportion of threatened species. Thus, we suggest these species may be more threatened than currently depicted in the Red List and would benefit from reassessment. Similar attention should be given to all species that moved to a more threatened category in our assessment (S1 Data). We recommend a strong precautionary approach in translating such disparities into conservation action.

Other than differences in range sizes between GARD and IUCN datasets, misclassifications of species as less threatened than assessed by the IUCN may be due to species meeting Red List criteria other than B, as their exclusion led to higher model accuracy. These criteria are mostly based on data on population sizes and trends, which are unavailable for most reptile species. Population dynamics are difficult to approximate using remotely sensed predictors [36] such as the ones used in most automated assessment methods. Excluding species classified as threatened under non-B criteria from model training caused their extinction risk to be severely underestimated (S4 Table). This highlights that the inclusion of population size and trend data in the model can only increase the level of predicted extinction risk compared to the result expected under criterion B only, mimicking the IUCN assessment process.

Nevertheless, most of our modeled classifications (for assessed species) are the same as the IUCN ones (94%, 6,112 of 6,520). The modeled assessments we obtained can be used to identify priorities for assessment of NE species, with species estimated to be at higher risk requiring more urgent assessment. Likewise, previously assessed species, which our method identified as being at higher extinction risk than their current IUCN category indicates, should be priority candidates for reassessment [25], especially in the case of species previously categorized as DD, as their current assessment does not allow their prioritization in conservation efforts. A major obstacle for the implementation of correlative automated assessment methods, such as the one we present, is the lack of explicit parameters to justify the assessment under existing criteria [25]. To overcome this obstacle, we propose the IUCN consider the creation of a parallel listing for automated assessments, to be displayed alongside IUCN assessments with clear indication of the provisional, modeled, status of the assessment. We recognize that the creation of this new feature is not a simple endeavor but suggest it could be highly beneficial for the IUCN Red List. As automated methods become more easily available and precise, they offer an opportunity that should not be ignored for advancing the conservation of neglected (or newly described [37]) taxa and regions. Moreover, our provisional assessments and method can be used in regional red lists, which have more flexible guidelines.

We applied our methods to all DD and NE reptiles globally. In practice, our method can also be applied to regional- and country-level assessments. This is the scale at which national red lists, which support many country-level conservation decisions, are made [38]. Nevertheless, in some regions, challenges, such as lack of resources or standardized methods for regional assessments, are especially salient [39]. Provisional assessments provided by automated methods such as ours can also be used to inform conservation policy and action on DD and NE species, which are currently often given little weight, if any. We recommend that the use of these provisional categories in conservation will be aligned with expert input, especially for species in borderline categories (VU and NT), for which the automated assessment was less reliable.

### Predictions for data deficient and not evaluated species

Our results suggest DD species are more likely to be threatened than categorized species, adding to growing evidence in that regard [8,14,17,40–42], but unlike previous automated assessments for reptiles [19,28]. However, it is important to note that previous assessments have drawn on different datasets, both with respect to predictors used and level of extinction risk, as range maps and extinction risk categories have since been updated. We further found that NE reptiles (similar to DD species) are more likely to be threatened than categorized species—supporting the urgency of previous calls for a comprehensive reptile assessment [9]. Our method relies on extent of occurrence maps, which were used as a hierarchical classifier in modeling. Non-DD-assessed species have an extent of occurrence that is 16% larger, on average, than DD and NE species (F-value: 6.93, *p*-value: 0.009). For NE species this may be caused by them being recently described (i.e., later than a workshop on the fauna of the area they inhabit was conducted) and thus having small extent of occurrence. Taxonomic revision resulting in species splits will also give rise to NE species with small extents of occurrence. With such alarmingly high levels of predicted threat, we recommend that decision-makers take a cautious stance and assign DD and NE species similar priority as threatened species, unless evidence to the contrary is available (e.g., having been assigned a nonthreatened category by an automated assessment).

DD species may have incomplete distribution records or suffer from taxonomic uncertainties (although only 69 of the 1,083 DD species examined here were classified as such due to taxonomic uncertainty), which might cause their ranges to be underestimated. On the other hand, many truly rare and small-ranged species lack information to be assigned an extinction risk category. It is useful to provide DD species with provisional assessments because they often cannot be included in conservation prioritization [42]. Thus, it is safer to assume that DD species indeed have the ranges from which they are presently known, rather than risking leaving very threatened species in an unprioritizable category [8].

### Phylogenetic and spatial patterns

Our results revealed an overall decrease in the proportion of threatened turtle species after the addition of our predictions for DD and NE species (Fig 2). This could be due to the more complete assessment of turtles than of squamates. Data on population sizes and trends are much more readily available for testudines than for squamates [43]. Only 19% of squamates were classified as threatened based (at least in part) on criteria other than B—compared to 83% of turtles. The proportion of threatened species tended to increase in some squamate groups, especially in small, fossorial, rare, and endemic taxa (Fig 2, S9 Table), which is consistent with previously reported patterns of data deficiency [9], or possibly caused by underestimation of their ranges. Our method is thus better suited for data-poor clades than for extremely data-rich ones. The latter have already been assessed or are easy to assess, but the former comprise most of global biodiversity. Thus, our method could be especially useful for other data-poor and underassessed groups, such as most invertebrate clades.

Our results suggest that the world’s unknown and rich biodiversity is at even greater risk than previously perceived. This finding adds to accumulating evidence that geographical and phylogenetic patterns of extinction risk and knowledge gaps are mostly congruent [10]. We further found that the proportion of threatened species increases in most ecoregions in the Americas, Australia, and Madagascar but decreases in most of Africa and Eurasia. This could be driven by a taxonomic effect, as many of the families predicted to increase in proportion of threatened species are especially diverse in the Americas, Australia, and Madagascar (e.g., Dactyloidae, Diplodactylidae, Dipsadidae, Elapidae, Phrynosomatidae, and Scincidae; Fig 2). Assessments of regions and taxa we identified as likely to be more threatened should be given increased attention in new assessments and conservation planning.

### Effect of assessor/reviewer identities on predictions

Our models achieved high levels of accuracy even without accounting for assessor/reviewer effects (Table 1). Nonetheless, the composition of assessors may greatly influence predictions across all categories (Figs 4A and S3 and S8 Table). A possible explanation for this pattern is that such effects could be implicitly accounted for in spatial and phylogenetic autocorrelation since assessors usually assess only particular taxa and locations (Table 1). For example, if a group of assessors worked mostly on assessment of South American turtles, the biases they introduce might be accounted by the spatial dependency associated with South America and phylogenetic dependency associated with Testudines.

For all realms except Oceania, we found assessor and reviewer identities affected IUCN assessments. The effect of permuting assessor/reviewer identities suggested that observed assessments were similar to those expected if all species were evaluated by the most pessimistic assessors/reviewers in Madagascar and the Nearctic realms. The lack of effects for Oceania (Fig 4B, S12 Table) is likely due to the small number of species in this realm and the few people assessing them. Several recommendations have been made to address assessor bias, including the need for thorough documentation and divulgation of contentious assessments, so they can be used for training and guideline refinement, and training assessors, specifically addressing handling uncertainty and assessor’s attitudes to risk [12,26]. We further recommend that the IUCN, and local or regional agencies wishing to assess extinction risk of species or populations, (1) conduct regular automated assessments of previously assessed species, followed by examination of discrepant cases and reassessment if necessary; (2) create a new parallel listing specifically tailored to provisional automated assessments, as long as the provisional status of the assessment is always clearly indicated (as mentioned above); and (3) recommend that data scientists are present during the assessment process, for the production and interpretation of analytical inputs such as automated assessments. This last recommendation is important as data science becomes an increasingly integral and important part of ecology and conservation [44,45]. Training ecologists in data science is the way forward for more efficient environmental science and conservation [46]. It is thus reasonable to expect that, in the near future, many volunteer assessors will have the necessary expertise to employ emergent automated assessment methods, but it is also crucial that developers make their methods easier to use, integrating them with available user interface platforms [25]. Short-term solutions could include making data scientists from within the IUCN network, and specifically within the IUCN Red List Partnership, available for consultation when needed.

We also recommend, as further research avenues, the development of (1) analytical methods to identify which assessment criteria and subcriteria are more subject to ambiguities, and how they can be refined; (2) applications for quick automated assessments using methods such as the one proposed here; and (3) automated assessment methods specifically geared toward modeling population sizes and trends (e.g., based on spatial distribution of threats such as land use changes, climate change, invasive species ranges, and hotspots of wildlife trade), to evaluate species using criteria other than B.

We have shown that accurate predictions can be made without explicitly accounting for assessor/reviewer effects. Previous automated assessments, which reported high levels of accuracy without accounting for assessor/reviewer effects, showed much lower accuracy when their predictions were confronted with manual assessments [28]. Biases from past assessments can be indirectly captured by algorithms and be accurately incorporated in predictions, but biases from future assessments could fall outside the scope of the training data. The contingency of manual assessments on assessor identities makes automated assessments more reliable, but those are also subject to many sources of uncertainty [47,48]. Moreover, since automated methods are trained using previous manual assessments, they risk carrying over the biases of past assessors. Automated methods that explicitly incorporate uncertainty into their predictions (e.g., [22]) are a promising avenue for future development, and they should explicitly account for assessor/reviewer effects. Overall, automated assessment can be a useful tool for provisional prioritization and assessment acceleration but should be viewed critically.

## Conclusions

We show that, with the inclusion of estimates for DD and NE species, reptiles globally emerge as more threatened than the IUCN Red List currently depicts. This underestimation is widespread across space and phylogeny. Our automated assessments accurately captured the extinction risk categories and could be widely used for generating provisional assessments for numerous taxa awaiting assessments. We nonetheless recommend that special attention is paid to population declines, which are less well captured by our model and result in it being conservative in assigning extinction risk categories. From a precautionary principle perspective, our results also support the notion that DD and NE should be candidates for increased conservation efforts until they are assigned a proper extinction risk category as they are approximately 30% more likely to be threatened than the other assessed species (27% versus 21%). While IUCN assessments will continue to be the gold standard for categorizing species threat, we recommend caution is necessary and that assessor/reviewer effects should be considered when using them. Altogether, our models predict that the state of reptile conservation is far worse than currently estimated and that immediate action is necessary to avoid the disappearance of reptile biodiversity.

## Materials and methods

### Data acquisition

We obtained distribution estimates of 10,889 terrestrial and freshwater reptile species (94% of the 11,570 currently recognized species) from an updated version of the Global Assessment of Reptile Distributions (GARD 1.7—Data deposited in the Dryad repository: https://doi.org/10.5061/dryad.9cnp5hqmb [49,50]). We extracted summary values for a suite of parameters obtained using the overlap of each species’ range with 5 classes of remotely sensed predictors. These include climate (76 features), human encroachment (45 features), biogeography (26 features), topography (9 features), ecosystem productivity (8 features), as well as the latitudinal centroid of each species’ distribution. Predictors and metadata are summarized in S1 Data. We added to these predictors species-level data on body mass and insularity assembled from the literature as part of the GARD initiative ([51]; see S1 Data). As other biological attributes are harder to come by (and consequently had a lot of missing values for our reptile species), we only included body mass as a species-level biological attribute. We used these data, together with measures of spatial and phylogenetic autocorrelation, and assessor and reviewer effects to model IUCN extinction risk categories using a recent gradient boosting algorithm (details below). While we used the best available data sources, with the most complete coverage, there might still be geographical biases in their precision. Such biases are likely to occur in any exploration of such a wide scope and we believe they do not detract from our method. We set aside 20% of species for validation. We used the 15 March 2021 IUCN reptile assessments [1]. All datasets were standardized to the taxonomy of the March 2021 version of the Reptile Database [52], with the input of experts from the GARD initiative. All analysis were conducted in R 4.0.3 [53].

### Incorporating spatial and phylogenetic autocorrelation

We used Moran’s Eigenvector Maps and Phylogenetic Eigenvector Maps to represent spatial and phylogenetic structure in our models [54,55]. The main advantage of these techniques is that they can be incorporated in modern machine learning methods, such as XGBoost [29] (description below). Eigenvector methods have been criticized for requiring the omission of part of the autocorrelation structure and not explicitly incorporating an evolutionary model [13,56]. Some of these critiques have since been resolved [55] and are less relevant in our case as we simply use eigenvectors as proxies for broad scale predictors of extinction risk (see also [57]).

We used the GARD distribution dataset to calculate Moran’s eigenvectors, employing R package “adespatial” [58]. We intersected species distribution polygons as neighbors and weighted the neighborhood matrix by inverse centroid distances calculated with function “nbdists” from package “spdep” [59]. To calculate phylogenetic eigenvectors, we used package “MPSEM” [60] and the phylogenies from Tonini and colleagues [31] for Squamata and Colston and colleagues [32] for Testudines and Crocodylia. We assumed a Brownian motion model of trait evolution. Species with distribution data, but no phylogenetic information (*n =* 167), were assigned an NA value for all phylogenetic eigenvectors. Squamata species were assigned NA value for the eigenvectors derived from the Testudines and Crocodylia tree, and Testudines and Crocodylia were assigned NA values for the eigenvectors derived from the Squamata tree. Positive eigenvalues are associated with autocorrelation at broader scales [54,55]. Since autocorrelation at small scales does not provide information on the entire structure [61], we used eigenvalues to reduce the number of eigenvectors, retaining only eigenvectors with eigenvalues larger than 10% of the eigenvalue of the first eigenvector. This left us with eigenvectors corresponding to autocorrelation structures deeper in the trees and across broader spatial scales. Following this procedure, we retained 236 spatial and 78 phylogenetic eigenvectors.

### Incorporating assessor and reviewer effects

We obtained the identity of 983 assessors and 192 reviewers for all evaluated reptiles on the 15 March 2021 using R package “rredlist” [62]. Many of these assessors and reviewers worked together on the assessments of different species in different combinations. To address this, we used an autocorrelative approach similar to our spatial autocorrelation detection/correction method, to incorporate potential assessor/reviewer effects in our models. We considered assessors/reviewers that worked together on a species assessment to be neighbors in the neighborhood matrix, with the number of species each pair assessed together as the weight of each pair’s association. Therefore, frequently associated assessors had more similar scores than those that associated occasionally. Assessors/reviewer scores were averaged for each eigenvector on each species. Therefore, species that were evaluated by a similar set of assessors/reviewers had more similar scores than species evaluated by more distinct sets of assessors/reviewers. We performed a priori selection based on eigenvalues, as described above, using the same thresholds, which resulted in 216 eigenvectors being retained for assessors and 39 for reviewers.

### Modeling threat

We used the XGBoost regularizing gradient boosting classification framework in our modeling of extinction risk categories. XGBoost is a recently developed machine learning algorithm that combines computational efficiency, versatility, and high levels of accuracy [29]. It is considered a state-of-the-art machine learning technique and is a popular choice for machine learning competitions [63]. Another advantage of XGBoost is its “Sparsity-aware Split Finding” algorithm, which enables effective classification of entries containing missing data [29]. XGBoost is also robust to imbalanced datasets [29], as is the case for reptile extinction risk categories, 72% of which are currently classified as LC [1]. We implemented this algorithm using the R package “xgboost” [64]. To compare model accuracy and efficiency across algorithms, we further fit a similar model using the AdaBoost algorithm [65], implemented in the R package “adabag” [66]. This approach obtained lower accuracy (see S1 Text).

**Fig 5:**
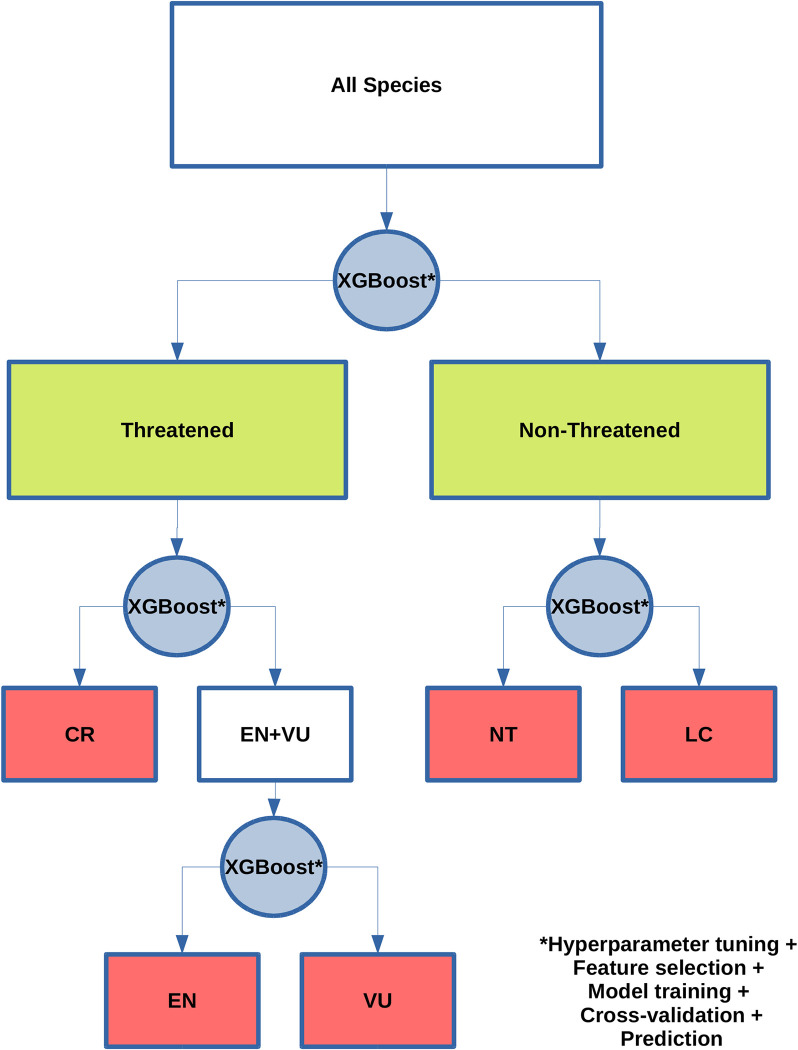
Flowchart for classification tasks in automated extinction risk assessment method, using the XGBoost algorithm [29]. Green boxes represent outcomes of the binary task and red boxes represent the outcome of the specific tasks. Steps taken for each classification task (blue circle) are indicated after the asterisk. CR, Critically Endangered; EN, Endangered; LC, Least Concern; NT, Near Threatened; VU, Vulnerable.

The range size of a species (as measured by extent of occurrence) can be used as an important a priori consideration for the assessment process, since most reptiles are assessed under criterion B. Consequently, we first separated species into the range size classes used in the IUCN Red List B criterion (over 20,000 km^2^, between 20,000 km^2^ and 5,000 km^2^, between 5,000 km^2^ and 100 km^2^, under 100 km^2^). This initial separation enabled different hyperparameter tuning, feature selection, and model fitting for each extent of occurrence class. Next, we used a decision tree (Fig 5) involving 4 hierarchical classification tasks for each extent of occurrence class: (1) separating threatened (CR, EN, and VU) from nonthreatened (NT and LC) species (binary classification); (2) separating CR species from other threatened species (EN and VU); (3) separating EN from VU in the remaining threatened species; and (4) separating NT from LC in the pool of nonthreatened species. We repeated this modeling approach after excluding threatened species not categorized under criterion B (360 species), to explore the amount of uncertainty introduced by the other Red List assessment criteria, which are less commonly used for reptiles. Hyperparameter tuning and feature selection was performed at each classification task (description in S1 Text). A detailed tutorial on how to reproduce our automated assessment method is available in S2 Text.

Since supervised machine learning methods, such as XGBoost, are primarily predictive, rather than mechanistic, features contributing to better predictions are not necessarily useful for making causal inferences [33]. Thus, we evaluated the contribution of phylogenetic eigenvectors, Moran’s eigenvectors, and assessor/reviewer effects by comparing models without these factors to models including them individually and in different combinations (i.e., a model with only autocorrelations and a model with autocorrelations and assessor/reviewer effects; Table 1). This allowed us to explore if their inclusion increases predictive power. We also fit a model for the dataset excluding threatened species assessed by criteria other than B, but without assessor/reviewer effects as predictors, to evaluate the importance of these features on this subset of assessments. We plotted the number of previously evaluated species that changed from threatened to nonthreatened categories and vice versa, for each biogeographical realm [67], to evaluate spatial biases in the model errors.

### Comparison with previous methods

We also compared the features of our model to previously published automated assessment methods (incorporation of spatial and phylogenetic autocorrelation, assessor bias, tolerance to missing data, and ability to predict specific IUCN categories). Beyond this, we implemented previous methods’ algorithms (when available), using our dataset of reptiles and predictors. These algorithms were Random Forest [17,18], and Neural Networks [19,20], implemented using the R packages “randomForest” [68] and “IUCNN” [20], respectively. We compared the prediction accuracy of these algorithms with the accuracy of our “Environment and body mass” model (Table 1) in the binary task of separating threatened and nonthreatened categories. We excluded spatial and phylogenetic eigenvectors for this analysis because the original implementation of the other methods we compared did not incorporate spatial and phylogenetic autocorrelation. Furthermore, phylogenetic eigenvectors contained a significant number of missing values, which are not tolerated by the Random Forest and Neural Networks implementations.

### Predictions for data deficient and not evaluated species

We used the model without assessor bias to estimate the extinction risk categories of DD and NE species. We used Pearson’s χ^2^ to test if the proportions of DD and NE species predicted to be threatened were significantly different from the assessed ones. We further tested if proportions predicted for each extinction risk category differ between DD, NE, and assessed species. We adjusted *p*-values using the false discovery rate correction [69].

### Phylogenetic and spatial patterns

We explored how our predictions for DD and NE species changed the overall proportion of threatened species across the reptile phylogeny [31,32], different ecoregions [67], and biogeographical realms. For our phylogenetic representation we compared the proportion of threatened species in each clade before and after the addition of our predictions for DD and NE species. We did this for all reptile families, as well as for each clade above the family level, and plotted the results along the branches of a composite phylogeny made from the trees of Tonini and colleagues [31] and Colston and colleagues [32].

We assigned species to ecoregions by intersecting species’ ranges from GARD 1.7 [49,50] with WWF terrestrial ecoregions of the world [67]. We compared the proportion of threatened species for each ecoregion, before and after the addition of predictions for DD and NE species. We also compared the percentage of threatened species before and after the inclusion of predictions for the eight terrestrial biogeographical realms: Afrotropics, Australasia, Indomalaya, Madagascar, Nearctic, Neotropics, Oceania, and Palearctic. Each species was assigned to all realms intersecting its range. The difference between proportions of threatened species in each biogeographical realm, before and after the inclusion of predictions, was tested using a χ^2^ test, with *p*-values corrected for multiple comparisons, using false discovery rate [69].

### Effect of assessor/reviewer identities on predictions

We evaluated the effect of assessor/reviewer identities on predictions for each extinction risk category. We sequentially permuted the assessor/reviewer eigenvector scores of each species to all other species, ran the modeling procedure described above, and retained the scores that resulted in least threatened (optimistic), and most threatened (pessimistic), categorizations. This procedure represents the potential results that would be obtained if the most “optimistic” and the most “pessimistic” group of assessors/reviewers assessed every species. This was done using the complete model using spatial and species-level predictors, spatial and phylogenetic autocorrelations, and assessor/reviewer effects, to minimize the effect of spatial and phylogenetic structure in assessor/reviewer assignments. We then tested if the resulting “optimistic” and “pessimistic” predictions were significantly different from the observed categories, and from each other, using χ^2^ tests, with *p*-values corrected for multiple comparisons, using false discovery rate. We performed a similar analysis to explore differences in assessor effects within each biogeographical realm for the binary classification task (of threatened/non-threatened categories).

### Dryad DOI

https://doi.org/10.5061/dryad.9cnp5hqmb.

## Supporting information

S1 Fig Contribution of feature classes to the predictive performance of automated assessment models classifying reptile species into IUCN extinction risk categories, for combinations of extent of occurrence class (columns, km^2^) and classification task (lines). The “Binary” task separates threatened (CR, EN, and VU) from nonthreatened categories (NT and LC). Features in each class had their contribution measures summed. “MEM” stands for Moran’s Eigenvector Maps, an indicator of spatial autocorrelation. “PEM” stands for Phylogenetic Eigenvector Maps, an indicator of phylogenetic autocorrelation. For the specific identity of features in each class, see S1 Data. The data underlying this figure can be found in S2 Data. CR, Critically Endangered; EN, Endangered; IUCN, International Union for Conservation of Nature; LC, Least Concern; NT, Near Threatened; VU, Vulnerable.
(TIFF)

S2 Fig Number of reptile species in 8 biogeographical realms that changed extinction risk category after application of an automated assessment method, compared to the IUCN categories, under 2 categorization schemes: (a) binary (threatened vs nonthreatened) categorization (b) specific IUCN categories (CR, EN, VU, NT, and LC). “Increases” indicates a species moved to a higher extinction risk category, “decreases” indicates it moved to a lower extinction risk category, and “remains” indicates extinction risk category stays the same. Y-axis is in log10 scale. The data underlying this figure can be found in S2 Data. AA, Australasian; AT, Afrotropical; CR, Critically Endangered; EN, Endangered; IM, Indomalayan; IUCN, International Union for Conservation of Nature; LC, Least Concern; MA, Madagascan; NA, Nearctic; NT, Neotropical; OC, Oceanian; PA, Palearctic; VU, Vulnerable.
(TIFF)

S3 Fig Heatmap of extinction risk category changes for different assessor bias scenarios. Upper off diagonal elements represent the movements of species from less threatened to more threatened categories (left to right), in the pessimistic scenario. Lower off diagonal elements represent the movements of species from less threatened to more threatened categories (right to left), in the optimistic scenario. Diagonal indicates the IUCN extinction risk categories species are moving to and from: CR, Critically Endangered; EN, Endangered; LC, Least Concern; NT, Near Threatened; VU, Vulnerable.
(TIFF)

S1 Table Accuracy metrics of automated assessment models classifying reptile species into IUCN extinction risk categories, under 8 different approaches. (1) complete model, accounting for spatial and phylogenetic autocorrelation and assessor/reviewer effects; (2) not accounting for spatial and phylogenetic autocorrelation or assessor/reviewer effects; (3) accounting for spatial autocorrelation; (4) accounting for phylogenetic autocorrelation; (5) accounting for spatial and phylogenetic autocorrelation; (6) accounting for assessor/reviewer effects; (7) accounting for spatial and phylogenetic autocorrelation and assessor/reviewer effects and excluding species categorized as threatened under criteria different from B; (8) accounting for spatial and phylogenetic autocorrelation and excluding species categorized as threatened under criteria different from B. “Binary” represents the separation of threatened (CR, EN, and VU) from nonthreatened categories (NT and LC). Remaining columns represent the predictive accuracy for assigning species to the 5 extinction risk categories: CR, Critically Endangered; EN, Endangered; LC, Least Concern; NT, Near Threatened; VU, Vulnerable.
(DOCX)

S2 Table Contribution of feature classes to the predictive performance of automated assessment models classifying reptile species into IUCN extinction risk categories, for combinations of extent of occurrence class (km^2^) and classification task. The “Binary” task separates threatened (CR, EN, and VU) from nonthreatened categories (NT and LC). Features in each class had their contribution measures summed. “MEM” stands for Moran’s Eigenvector Maps, an indicator of spatial autocorrelation. “PEM” stands for Phylogenetic Eigenvector Maps, an indicator of phylogentic autocorrelation. “Assessors” and “reviewers” stand for effects associated with the identity of assessors and reviewers that worked on each assessment. For the specific identity of features in each class, see S1 Data. CR, Critically Endangered; EN, Endangered; IUCN, International Union for Conservation of Nature; LC, Least Concern; NT, Near Threatened; VU, Vulnerable.
(DOCX)

S3 Table Optimal XGBoost hyperparameter configuration for each combination of classification tasks and extent of occurrence class. Parameters adjusted were as follows: learning rate (η), maximum tree depth (max_depth), minimum child weight (min_weight), row sampling (rowsample), column sampling (colsample), weight balancing (pos_weight), and 3 regularization parameters (γ, α, and λ). Hyperparameter tuning strategy described in S1 Text.
(DOCX)

S4 Table Number of reptile species classified as threatened under non-B criteria in each IUCN category before (rows) and after (columns) application of automated assessment method trained on B criteria species. IUCN, International Union for Conservation of Nature.
(DOCX)

S5 Table Accuracy metrics of 2 previously published automated assessment models for separating reptile species into threatened (CR, EN, and VU) and nonthreatened categories (NT and LC) IUCN extinction risk categories. Random Forest refers to the approach described by Bland and colleagues [17], and Neural Networks refers to the approach described by Zizka and colleagues [20]. CR, Critically Endangered; EN, Endangered; IUCN, International Union for Conservation of Nature; LC, Least Concern; NT, Near Threatened; VU, Vulnerable.
(DOCX)

S6 Table Comparison of automated assessment methods. Models are compared in their incorporation of spatial and phylogenetic autocorrelation, as well as their ability to account for assessor bias, including missing data and predicting specific IUCN categories. The method presented here is indicated as Caetano and colleagues [70]. IUCN, International Union for Conservation of Nature.
(DOCX)

S7 Table Pearson’s χ^2^ test statistics for comparisons of the proportion of reptile species assigned to each IUCN category between the actual assessments (Observed) and the predictions for DD and NE species, made using an automated assessment model. We adjusted *p*-values adjusted for false discovery rate. “Threatened” represents the proportion of species assigned a threatened category (CR, EN, and VU). Significant *p*-values are in bold. CR, Critically Endangered; DD, Data Deficient; EN, Endangered; LC, Least Concern; NE, Not Evaluated; NT, Near Threatened; VU, Vulnerable.
(DOCX)

S8 Table Number of reptile species in each IUCN category before (rows) and after (columns) application of automated assessment method. IUCN, International Union for Conservation of Nature.
(DOCX)

S9 Table Difference in the proportion of threatened species in reptile families before and after the addition of extinction risk estimates for DD and NE species, obtained from an automated assessment method. DD, Data Deficient; NE, Not Evaluated.
(DOCX)

S10 Table Pearson’s χ^2^ test statistics for comparisons of the proportion of threatened reptile species in 8 biogeographical realms, before and after the inclusion of predictions for DD and NE species, made using an automated assessment model. We adjusted *p*-values adjusted for false discovery rate. Significant *p*-values are in bold. DD, Data Deficient; NE, Not Evaluated.
(DOCX)

S11 Table Pearson’s χ^2^ test statistics for comparisons of the proportion of reptile species assigned to each IUCN category between the actual assessments (Observed) and the expected if the most optimist group of assessors assessed every species (Optimist) and if the most group pessimist assessed every species (Pessimist), estimated using an automated assessment model. We adjusted *p*-values adjusted for false discovery rate. “Threatened” represents the proportion of species assigned a threatened category (CR, EN, and VU). Significant *p*-values are in bold. CR, Critically Endangered; EN, Endangered; LC, Least Concern; NT, Near Threatened; VU, Vulnerable.
(DOCX)

S12 Table Pearson’s χ^2^ test statistics for comparisons of the proportion of threatened reptile species in 8 biogeographical realms between the actual assessments (Observed) and the expected if the most optimist group of assessors assessed every species (Optimist) and if the most group pessimist assessed every species (Pessimist), estimated using an automated assessment model. We adjusted *p*-values adjusted for false discovery rate.
(DOCX)

S1 Text Additional details on methods and results. This includes strategies used for hyperparameter tuning and feature selection and model accuracies resulting from the use of a different optimization criteria for hyperparameter optimization and feature selection (F1-score instead of AUC) and a different classification algorithm (AdaBoost instead of XGBoost).
(DOCX)

S2 Text Tutorial for reproducing automated assessment method. (PDF)

S1 File R code used for tutorial in S2 Text. (RMD)

S1 Data Metadata and additional results for automated assessment of world reptiles. This includes metadata regarding spatial predictors and species-level predictors. Also included are the results of feature selection for each classification task and the complete list of predicted categories for the world reptiles.
(XLSX)

S2 Data Data underlying manuscript figures. (XLSX)

## Abbreviations

CR: Critically Endangered
DD: Data Deficient
EN: Endangered
EW: Extinct in the Wild
EX: Extinct
IUCN: International Union for Conservation of Nature
LC: Least Concern
NE: Not Evaluated
NT: Near Threatened
VU: Vulnerable
