# Supplementary figures and images for: Automated assessment reveals that the extinction risk of reptiles is widely underestimated across space and phylogeny
Source: PLoS Biol. 2022 May 26;20(5):e3001544. doi: 10.1371/journal.pbio.3001544 (PMC9135251; doi:10.1371/journal.pbio.3001544)

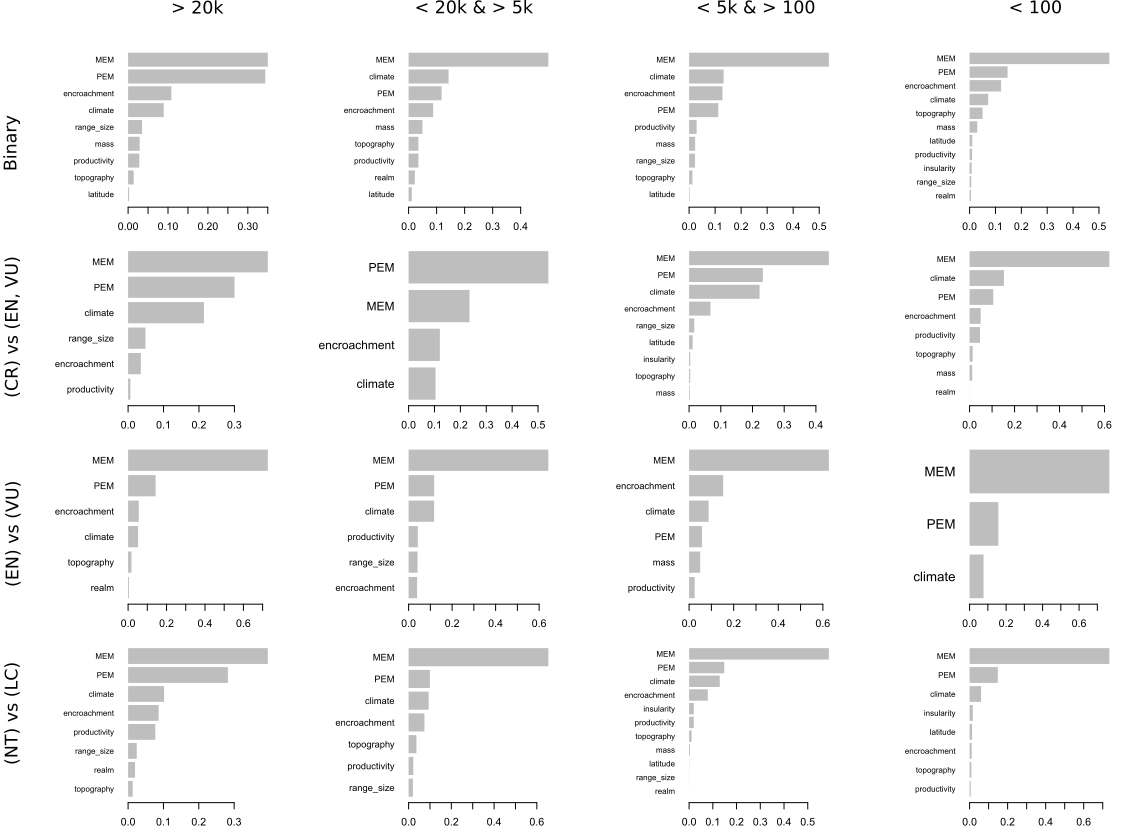

Supplement: S1 Fig — The “Binary” task separates threatened (CR, EN, and VU) from nonthreatened categories (NT and LC). Features in each class had their contribution measures summed. “MEM” stands for Moran’s Eigenvector Maps, an indicator of spatial autocorrelation. “PEM” stands for Phylogenetic Eigenvector Maps, an indicator of phylogenetic autocorrelation. For the specific identity of features in each class, see S1 Data. The data underlying this figure can be found in S2 Data. CR, Critically Endangered; EN, Endangered; IUCN, International Union for Conservation of Nature; LC, Least Concern; NT, Near Threatened; VU, Vulnerable. (TIFF) [file pbio.3001544.s001.tiff]

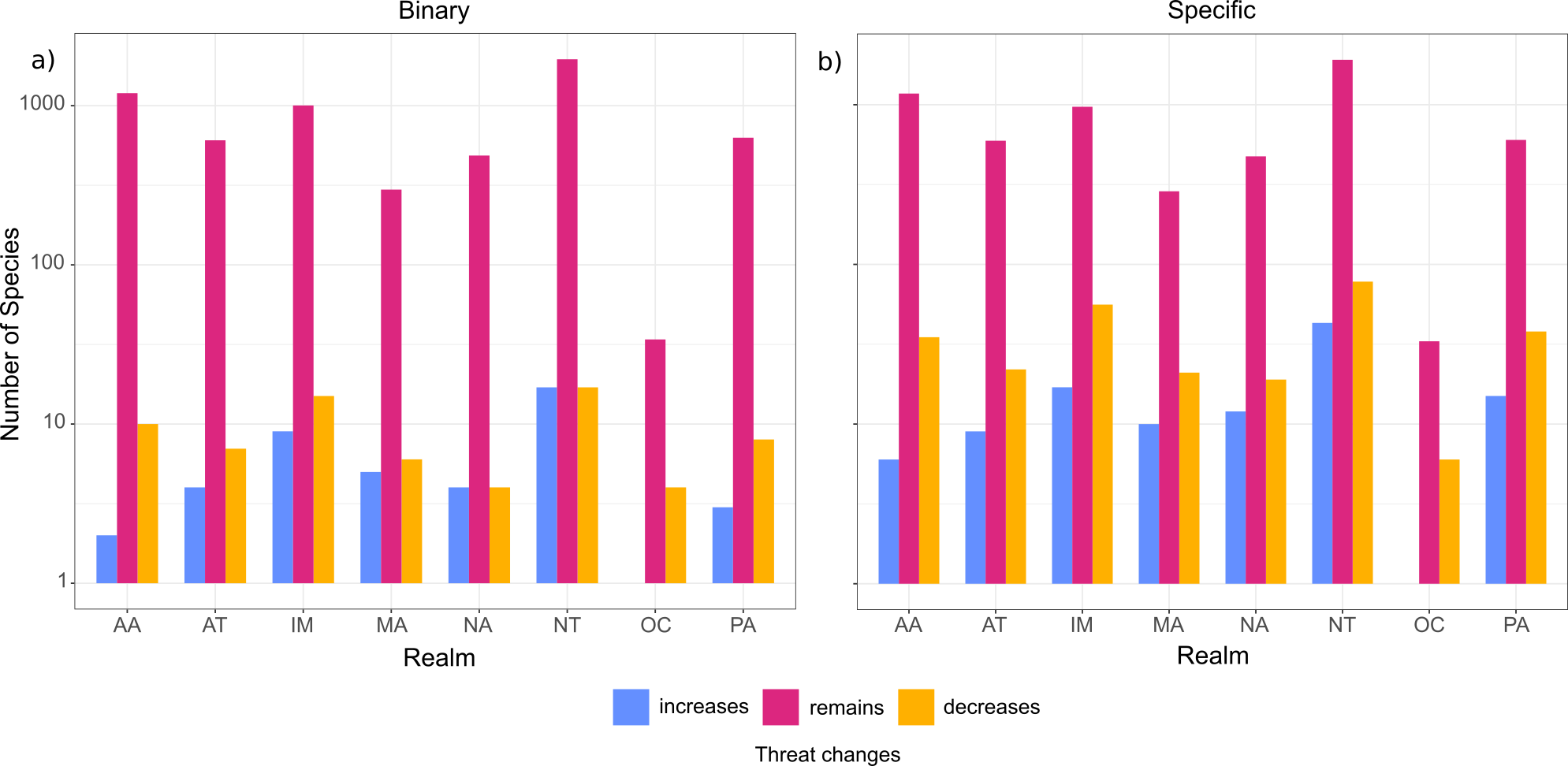

Supplement: S2 Fig — “Increases” indicates a species moved to a higher extinction risk category, “decreases” indicates it moved to a lower extinction risk category, and “remains” indicates extinction risk category stays the same. Y-axis is in log10 scale. The data underlying this figure can be found in S2 Data. AA, Australasian; AT, Afrotropical; CR, Critically Endangered; EN, Endangered; IM, Indomalayan; IUCN, International Union for Conservation of Nature; LC, Least Concern; MA, Madagascan; NA, Nearctic; NT, Neotropical; OC, Oceanian; PA, Palearctic; VU, Vulnerable. (TIFF) [file pbio.3001544.s002.tiff]

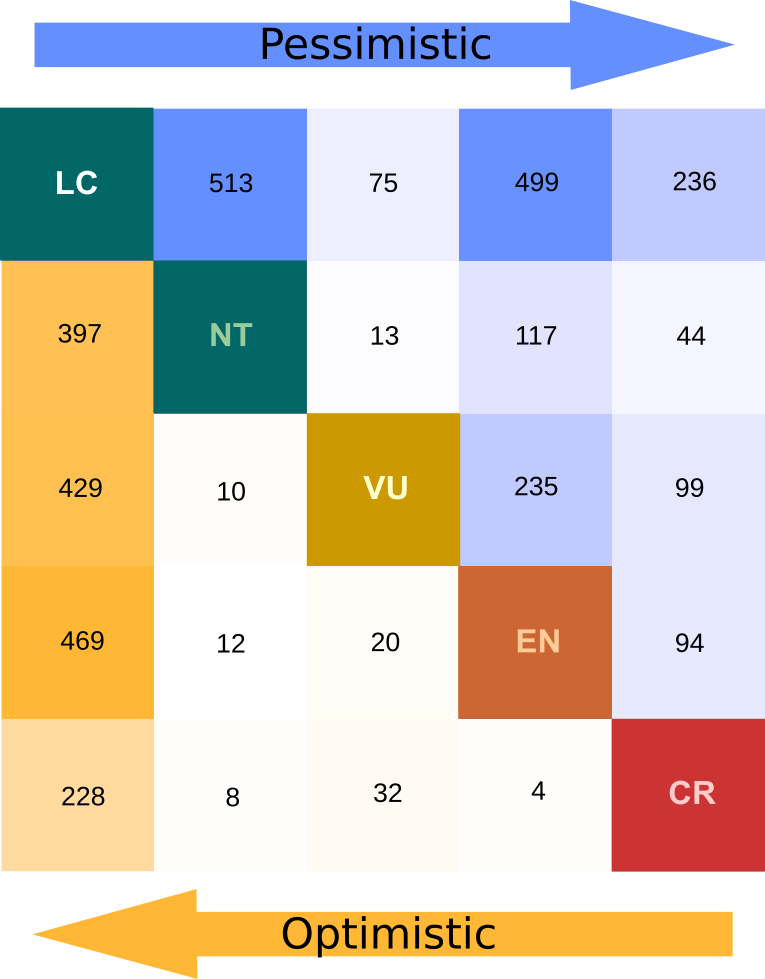

Supplement: S3 Fig — Upper off diagonal elements represent the movements of species from less threatened to more threatened categories (left to right), in the pessimistic scenario. Lower off diagonal elements represent the movements of species from less threatened to more threatened categories (right to left), in the optimistic scenario. Diagonal indicates the IUCN extinction risk categories species are moving to and from: CR, Critically Endangered; EN, Endangered; LC, Least Concern; NT, Near Threatened; VU, Vulnerable. (TIFF) [file pbio.3001544.s003.tiff]
