# Supplementary material for: Automated assessment reveals that the extinction risk of reptiles is widely underestimated across space and phylogeny
Source: PLoS Biol. 2022 May 26;20(5):e3001544. doi: 10.1371/journal.pbio.3001544 (PMC9135251; doi:10.1371/journal.pbio.3001544)
Supplement: S1 Table — (1) complete model, accounting for spatial and phylogenetic autocorrelation and assessor/reviewer effects; (2) not accounting for spatial and phylogenetic autocorrelation or assessor/reviewer effects; (3) accounting for spatial autocorrelation; (4) accounting for phylogenetic autocorrelation; (5) accounting for spatial and phylogenetic autocorrelation; (6) accounting for assessor/reviewer effects; (7) accounting for spatial and phylogenetic autocorrelation and assessor/reviewer effects and excluding species categorized as threatened under criteria different from B; (8) accounting for spatial and phylogenetic autocorrelation and excluding species categorized as threatened under criteria different from B. “Binary” represents the separation of threatened (CR, EN, and VU) from nonthreatened categories (NT and LC). Remaining columns represent the predictive accuracy for assigning species to the 5 extinction risk categories: CR, Critically Endangered; EN, Endangered; LC, Least Concern; NT, Near Threatened; VU, Vulnerable. (DOCX) [file pbio.3001544.s004.docx]

**S1 Table. Accuracy metrics of automated assessment models classifying reptile species into IUCN extinction risk categories, under eight different approaches**: 1) complete model, accounting for spatial and phylogenetic autocorrelation and assessor/reviewer effects, 2) not accounting for spatial and phylogenetic autocorrelation or assessor/reviewer effects, 3) accounting for spatial autocorrelation, 4) accounting for phylogenetic autocorrelation, 5) accounting for spatial and phylogenetic autocorrelation, 6) accounting for assessor/reviewer effects, 7) accounting for spatial and phylogenetic autocorrelation and assessor/reviewer effects and excluding species categorized as threatened under criteria different from B, 8) accounting for spatial and phylogenetic autocorrelation and excluding species categorized as threatened under criteria different from B. 'Binary' represents the separation of threatened (CR, EN and VU) from non-threatened categories (NT and LC). Remaining columns represent the predictive accuracy for assigning species to the five extinction risk categories: CR – Critically Endangered, EN – Endangered, VU – Vulnerable, NT – Near Threatened, LC – Least Concern.

|  | Binary | CR | EN | VU | NT | LC |
| --- | --- | --- | --- | --- | --- | --- |
| **Complete** | | | | | | |
| Sensitivity | 0.955 | 0.773 | 0.699 | 0.532 | 0.278 | 0.964 |
| Specificity | 0.711 | 0.997 | 0.976 | 0.977 | 0.983 | 0.691 |
| AUC | 0.833 | 0.885 | 0.837 | 0.755 | 0.631 | 0.828 |
| Precision | 0.925 | 0.927 | 0.731 | 0.649 | 0.556 | 0.890 |
| Recall | 0.955 | 0.773 | 0.699 | 0.532 | 0.278 | 0.964 |
| F1 | 0.940 | 0.843 | 0.715 | 0.585 | 0.370 | 0.925 |
| **Environment and body mass** | | | | | | |
| Sensitivity | 0.944 | 0.439 | 0.000 | 0.425 | 0.278 | 0.942 |
| Specificity | 0.623 | 0.913 | 0.986 | 0.972 | 0.973 | 0.636 |
| AUC | 0.784 | 0.676 | 0.493 | 0.699 | 0.625 | 0.789 |
| Precision | 0.904 | 0.213 | 0.000 | 0.540 | 0.431 | 0.870 |
| Recall | 0.944 | 0.439 | 0.000 | 0.425 | 0.278 | 0.942 |
| F1 | 0.924 | 0.287 | NA | 0.476 | 0.338 | 0.905 |
| **Assessor/reviewer effects** | | | | | | |
| Sensitivity | 0.951 | 0.652 | 0.664 | 0.468 | 0.389 | 0.949 |
| Specificity | 0.659 | 0.994 | 0.970 | 0.980 | 0.973 | 0.689 |
| AUC | 0.8053 | 0.823 | 0.817 | 0.724 | 0.681 | 0.818 |
| Precision | 0.913 | 0.843 | 0.676 | 0.647 | 0.515 | 0.887 |
| Recall | 0.951 | 0.652 | 0.664 | 0.468 | 0.389 | 0.949 |
| F1 | 0.932 | 0.735 | 0.670 | 0.543 | 0.443 | 0.917 |
| **Spatial** | | | | | | |
| Sensitivity | 0.948 | 0.727 | 0.611 | 0.425 | 0.300 | 0.948 |
| Specificity | 0.667 | 0.989 | 0.970 | 0.975 | 0.974 | 0.672 |
| AUC | 0.807 | 0.858 | 0.791 | 0.700 | 0.637 | 0.810 |
| Precision | 0.915 | 0.787 | 0.663 | 0.571 | 0.465 | 0.882 |
| Recall | 0.948 | 0.727 | 0.611 | 0.425 | 0.300 | 0.948 |
| F1 | 0.931 | 0.756 | 0.636 | 0.488 | 0.365 | 0.914 |
| **Phylogenetic** | | | | | | |
| Sensitivity | 0.944 | 0.742 | 0.637 | 0.289 | 0.457 | 0.943 |
| Specificity | 0.656 | 0.993 | 0.971 | 0.980 | 0.975 | 0.642 |
| AUC | 0.800 | 0.868 | 0.804 | 0.634 | 0.716 | 0.793 |
| Precision | 0.912 | 0.860 | 0.679 | 0.520 | 0.589 | 0.872 |
| Recall | 0.944 | 0.742 | 0.637 | 0.289 | 0.457 | 0.943 |
| F1 | 0.928 | 0.797 | 0.657 | 0.371 | 0.515 | 0.906 |
| **Spatial-phylogenetic** | | | | | | |
| Sensitivity | 0.952 | 0.621 | 0.726 | 0.278 | 0.532 | 0.950 |
| Specificity | 0.703 | 0.995 | 0.969 | 0.976 | 0.979 | 0.683 |
| AUC | 0.828 | 0.808 | 0.847 | 0.627 | 0.756 | 0.816 |
| Precision | 0.923 | 0.872 | 0.689 | 0.463 | 0.667 | 0.886 |
| Recall | 0.952 | 0.621 | 0.726 | 0.278 | 0.532 | 0.950 |
| F1 | 0.938 | 0.726 | 0.707 | 0.347 | 0.592 | 0.917 |
|  | | | | | | |
| **Complete - Criterion B** | | | | | | |
| Sensitivity | 0.969 | 0.822 | 0.663 | 0.597 | 0.300 | 0.972 |
| Specificity | 0.708 | 0.990 | 0.986 | 0.991 | 0.988 | 0.651 |
| AUC | 0.838 | 0.906 | 0.824 | 0.794 | 0.644 | 0.811 |
| Precision | 0.944 | 0.755 | 0.797 | 0.787 | 0.658 | 0.899 |
| Recall | 0.9688 | 0.822 | 0.663 | 0.597 | 0.300 | 0.972 |
| F1 | 0.8384 | 0.787 | 0.724 | 0.679 | 0.412 | 0.934 |
| **Spatial-phylogenetic - Criterion B** | | | | | | |
| Sensitivity | 0.972 | 0.622 | 0.632 | 0.516 | 0.322 | 0.966 |
| Specificity | 0.629 | 0.992 | 0.987 | 0.991 | 0.975 | 0.620 |
| AUC | 0.800 | 0.807 | 0.809 | 0.753 | 0.649 | 0.793 |
| Precision | 0.930 | 0.737 | 0.800 | 0.744 | 0.509 | 0.891 |
| Recall | 0.972 | 0.622 | 0.632 | 0.516 | 0.322 | 0.966 |
| F1 | 0.950 | 0.675 | 0.706 | 0.609 | 0.395 | 0.927 |
