# Supplementary material for: Automated assessment reveals that the extinction risk of reptiles is widely underestimated across space and phylogeny
Source: PLoS Biol. 2022 May 26;20(5):e3001544. doi: 10.1371/journal.pbio.3001544 (PMC9135251; doi:10.1371/journal.pbio.3001544)
Supplement: S2 Table — The “Binary” task separates threatened (CR, EN, and VU) from nonthreatened categories (NT and LC). Features in each class had their contribution measures summed. “MEM” stands for Moran’s Eigenvector Maps, an indicator of spatial autocorrelation. “PEM” stands for Phylogenetic Eigenvector Maps, an indicator of phylogentic autocorrelation. “Assessors” and “reviewers” stand for effects associated with the identity of assessors and reviewers that worked on each assessment. For the specific identity of features in each class, see S1 Data. CR, Critically Endangered; EN, Endangered; IUCN, International Union for Conservation of Nature; LC, Least Concern; NT, Near Threatened; VU, Vulnerable. (DOCX) [file pbio.3001544.s005.docx]

**S2 Table. Contribution of feature classes to the predictive performance of automated assessment models classifying reptile species into IUCN extinction risk categories, for combinations of extent of occurrence class (km^2^) and classification task.** The 'Binary' task separates threatened (CR, EN and VU) from non-threatened categories (NT and LC). CR – Critically Endangered, EN – Endangered, VU – Vulnerable, NT – Near Threatened, LC – Least Concern. Features in each class had their contribution measures summed. 'MEM' stands for Moran’s Eigenvector Maps, an indicator of spatial autocorrelation. 'PEM' stands for Phylogenetic Eigenvector Maps, an indicator of phylogentic autocorrelation. 'Assessors' and 'reviewers' stand for effects associated with the identity of assessors and reviewers that worked on each assessment. For the specific identity of features in each class, see S1 Data.

| Range size class | > 20.000 km^2^ | | | | < 20.000 km^2^ & > 5000 km^2^ | | | | < 5000 km^2^ & > 100 km^2^ | | | | < 100 km^2^ | | | | Rank |
| --- | --- | --- | --- | --- | --- | --- | --- | --- | --- | --- | --- | --- | --- | --- | --- | --- | --- |
| Classification task | Binary | CR vs EN+VU | EN vs VU | NT vs LC | Binary | CR vs EN+VU | EN vs VU | NT vs LC | Binary | CR vs EN+VU | EN vs VU | NT vs LC | Binary | CR vs EN+VU | EN vs VU | NT vs LC |  |
| Complete model | | | | | | | | | | | | | | | | | |
| MEM | 0.328 | 0.296 | 0.063 | 0.174 | 0.081 | 0.471 | 0.135 | 0.098 | 0.083 | 0.219 | 0.072 | 0.116 | 0.100 | 0.118 | 0.127 | 0.025 | 1 |
| PEM | 0.223 | 0.312 | 0.538 | 0.258 | 0.319 | 0.529 | 0.545 | 0.355 | 0.343 | 0.525 | 0.557 | 0.498 | 0.356 | 0.453 | 0.561 | 0.710 | 3 |
| assessors | 0.192 | 0.145 | 0.237 | 0.212 | 0.342 | - | 0.211 | 0.289 | 0.328 | 0.065 | 0.189 | 0.234 | 0.319 | 0.190 | 0.198 | 0.200 | 2 |
| reviewers | 0.081 | - | 0.058 | 0.105 | 0.082 | - | 0.090 | 0.096 | 0.080 | 0.039 | 0.086 | 0.065 | 0.085 | 0.079 | 0.011 | - | 7 |
| latitude | 0.066 | 0.120 | 0.015 | 0.087 | 0.054 | - | - | 0.085 | 0.072 | 0.042 | 0.032 | 0.043 | 0.089 | 0.083 | 0.070 | 0.025 | 12 |
| insularity | 0.032 | - | 0.013 | 0.041 | 0.007 | - | - | 0.001 | 0.021 | 0.072 | - | - | - | 0.003 | - | - | 6 |
| topography | 0.032 | - | - | 0.018 | 0.024 | - | - | 0.001 | 0.011 | - | 0.040 | 0.011 | 0.005 | - | - | - | 8 |
| range size | 0.020 | - | 0.005 | 0.016 | 0.009 | - | - | 0.013 | 0.029 | - | 0.007 | - | 0.014 | 0.022 | 0.017 | - | 10 |
| biogeography | 0.015 | 0.127 | 0.023 | 0.065 | 0.032 | - | - | 0.031 | 0.013 | - | 0.016 | - | 0.009 | 0.003 | - | - | 13 |
| climate | 0.010 | - | 0.048 | 0.023 | 0.016 | - | 0.019 | 0.029 | 0.015 | 0.039 | - | 0.010 | 0.021 | 0.013 | 0.007 | - | 4 |
| encroachment | - | - | - | - | 0.034 | - | - | 0.000 | 0.005 | - | - | - | - | 0.008 | - | - | 5 |
| Range size class | > 20.000 km^2^ | | | | < 20.000 km^2^ & > 5000 km^2^ | | | | < 5000 km^2^ & > 100 km^2^ | | | | < 100 km^2^ | | | | Rank |
| Classification task | Binary | CR vs EN+VU | EN vs VU | NT vs LC | Binary | CR vs EN+VU | EN vs VU | NT vs LC | Binary | CR vs EN+VU | EN vs VU | NT vs LC | Binary | CR vs EN+VU | EN vs VU | NT vs LC |  |
| productivity | - | - | - | - | - | - | - | - | 0.001 | - | - | 0.003 | - | - | 0.010 | - | 9 |
| body mass | - | - | - | - | - | - | - | - | - | - | - | 0.020 | 0.003 | 0.028 | - | 0.040 | 11 |
| No assessor/reviewer effects | | | | | | | | | | | | | | | | | |
| MEM | 0.350 | 0.394 | 0.727 | 0.395 | 0.499 | 0.235 | 0.642 | 0.654 | 0.537 | 0.441 | 0.627 | 0.589 | 0.540 | 0.621 | 0.766 | 0.735 | 1 |
| PEM | 0.344 | 0.300 | 0.143 | 0.282 | 0.118 | 0.540 | 0.118 | 0.100 | 0.112 | 0.233 | 0.058 | 0.148 | 0.147 | 0.105 | 0.158 | 0.149 | 2 |
| latitude | 0.108 | 0.036 | 0.056 | 0.086 | 0.088 | 0.121 | 0.039 | 0.074 | 0.128 | 0.067 | 0.153 | 0.079 | 0.122 | 0.049 | - | 0.011 | 10 |
| insularity | 0.089 | 0.214 | 0.052 | 0.102 | 0.143 | 0.104 | 0.117 | 0.094 | 0.133 | 0.222 | 0.088 | 0.129 | 0.072 | 0.153 | 0.077 | 0.060 | 5 |
| topography | 0.035 | 0.049 | - | 0.024 | - | - | 0.041 | 0.020 | 0.023 | 0.016 | - | 0.001 | 0.006 | - | - | - | 8 |
| range size | 0.029 | - | - | - | 0.049 | - | - | - | 0.023 | 0.002 | 0.050 | 0.004 | 0.030 | 0.012 | - | - | 9 |
| biogeography | 0.028 | 0.006 | - | 0.077 | 0.035 | - | 0.043 | 0.022 | 0.029 | - | 0.025 | 0.020 | 0.010 | 0.046 | - | 0.006 | 11 |
| climate | 0.014 | - | 0.018 | 0.013 | 0.035 | - | - | 0.037 | 0.013 | 0.003 | - | 0.010 | 0.050 | 0.013 | - | 0.009 | 3 |
| encroachment | 0.003 | - | - | - | 0.011 | - | - | - | 0.002 | 0.011 | - | 0.001 | 0.010 | - | - | 0.013 | 4 |
| productivity | - | - | 0.005 | 0.020 | 0.022 | - | - | - | - | - | - | 0.000 | 0.005 | 0.000 | - | - | 7 |
| body mass | - | - | - | - | - | - | - | - | - | 0.004 | - | 0.020 | 0.008 | - | - | 0.017 | 6 |
