# Supplementary material for: Automated assessment reveals that the extinction risk of reptiles is widely underestimated across space and phylogeny
Source: PLoS Biol. 2022 May 26;20(5):e3001544. doi: 10.1371/journal.pbio.3001544 (PMC9135251; doi:10.1371/journal.pbio.3001544)
Supplement: S3 Table — Parameters adjusted were as follows: learning rate (η), maximum tree depth (max_depth), minimum child weight (min_weight), row sampling (rowsample), column sampling (colsample), weight balancing (pos_weight), and 3 regularization parameters (γ, α, and λ). Hyperparameter tuning strategy described in S1 Text. (DOCX) [file pbio.3001544.s006.docx]

**S3 Table. Optimal XGBoost hyperparameter configuration for each combination of classification tasks and extent of occurrence class.** Parameters adjusted were: learning rate (η), maximum tree depth (max_depth), minimum child weight (min_weight), row sampling (rowsample), column sampling (colsample), weight balancing (pos_weight), and three regularization parameters (γ, α, λ). Hyperparameter tuning strategy described in S1 Text.

| Task/Range Class | η | max_depth | min_weight | rowsample | colsample | pos_weight | γ | α | λ |
| --- | --- | --- | --- | --- | --- | --- | --- | --- | --- |
| Binary | | | | | | | | | |
| > 20k | 0.4 | 7 | 1 | 1 | 1 | 1 | 0 | 0 | 1.1 |
| < 20k & > 5k | 0.4 | 3 | 1 | 1 | 1 | 1 | 0 | 0 | 1 |
| < 5k & >100 | 0.3 | 6 | 1 | 1 | 1 | 1 | 0.2 | 0 | 1 |
| < 100 | 0.3 | 6 | 1 | 1 | 1 | 1 | 0 | 0 | 1 |
| (CR) vs (EN, VU) | | | | | | | | | |
| > 20k | 0.7 | 4 | 1.8 | 1 | 0.8 | 1 | 0 | 0.8 | 1 |
| < 20k & > 5k | 1 | 6 | 1 | 1 | 0.4 | 1 | 0 | 0 | 1 |
| < 5k & >100 | 0.1 | 6 | 0.2 | 1 | 0.3 | 0.3 | 0 | 0 | 1 |
| < 100 | 0.4 | 4 | 0.1 | 0.7 | <0.001 | 1 | 0 | 0 | 1.2 |
| (EN) vs (VU) | | | | | | | | | |
| > 20k | 0.2 | 2 | 0.5 | 0.5 | 1 | 1 | 0 | 0 | 1 |
| < 20k & > 5k | 0.2 | 3 | 0.9 | 1 | 1 | 1.5 | 0 | 0 | 1 |
| < 5k & >100 | 1 | 3 | 1.7 | 0.8 | 1 | 1 | 0.1 | 0 | 1 |
| < 100 | 0.7 | 6 | 0.2 | 1 | 1 | 1.1 | 0 | 0 | 0.8 |
| (NT) vs (LC) | | | | | | | | | |
| > 20k | 0.6 | 2 | 1.5 | 0.4 | 0.6 | 1 | 0 | 0 | 0.1 |
| < 20k & > 5k | 0.4 | 4 | 1.1 | 1 | 1 | 1.5 | 0 | 0 | 1.3 |
| < 5k & >100 | 0.1 | 7 | 1 | 1 | 0.2 | 1 | 0 | 0 | 1 |
| < 100 | 0.5 | 6 | 1 | 1 | 0.9 | 1.3 | 0 | 0.1 | 0.5 |
