# Supplementary material for: Automated assessment reveals that the extinction risk of reptiles is widely underestimated across space and phylogeny
Source: PLoS Biol. 2022 May 26;20(5):e3001544. doi: 10.1371/journal.pbio.3001544 (PMC9135251; doi:10.1371/journal.pbio.3001544)
Supplement: S4 Table — IUCN, International Union for Conservation of Nature. (DOCX) [file pbio.3001544.s007.docx]

**S4 Table.** **Number of reptile species classified as threatened under non-B criteria in each IUCN category before (rows) and after (columns) application of automated assessment method trained on B criteria species.**

|  | CR | EN | VU | NT | LC | **Total** |
| --- | --- | --- | --- | --- | --- | --- |
| CR | 4 | 7 | 10 | 24 | 21 | **66** |
| EN | 3 | 3 | 4 | 25 | 11 | **46** |
| VU | 21 | 34 | 5 | 47 | 141 | **248** |
| **Total** | **28** | **44** | **19** | **96** | **173** | **360** |
