# Supplementary material for: Automated assessment reveals that the extinction risk of reptiles is widely underestimated across space and phylogeny
Source: PLoS Biol. 2022 May 26;20(5):e3001544. doi: 10.1371/journal.pbio.3001544 (PMC9135251; doi:10.1371/journal.pbio.3001544)
Supplement: S5 Table — Random Forest refers to the approach described by Bland and colleagues [17], and Neural Networks refers to the approach described by Zizka and colleagues [20]. CR, Critically Endangered; EN, Endangered; IUCN, International Union for Conservation of Nature; LC, Least Concern; NT, Near Threatened; VU, Vulnerable. (DOCX) [file pbio.3001544.s008.docx]

**S5 Table. Accuracy metrics of two previously published automated assessment models for separating reptile species into threatened (CR, EN and VU) and non-threatened categories (NT and LC) IUCN extinction risk categories.** Random Forest refers to the approach described by Bland et al, 2015 [1], and Neural networks refers to the approach described by Zizka et al, 2020 [2].

|  | Random Forest | Neural Networks |
| --- | --- | --- |
| Accuracy | 0.849 | 0.788 |
| Sensitivity | 0.947 | 0.998 |
| Specificity | 0.482 | 0.004 |
| AUC | 0.714 | 0.501 |
| Precision | 0.873 | 0.789 |
| Recall | 0.947 | 0.998 |
| F1 | 0.908 | 0.881 |

**References**

1. Bland LM, Collen BEN, Orme CDL, Bielby JON. Predicting the conservation status of data-deficient species. Conservation Biology. 2015;29: 250–259.
2. Zizka A, Silvestro D, Vitt P, Knight TM. Automated conservation assessment of the orchid family with deep learning. Conservation Biology. 2021;35: 897–908.
