# Supplementary material for: Automated assessment reveals that the extinction risk of reptiles is widely underestimated across space and phylogeny
Source: PLoS Biol. 2022 May 26;20(5):e3001544. doi: 10.1371/journal.pbio.3001544 (PMC9135251; doi:10.1371/journal.pbio.3001544)
Supplement: S6 Table — Models are compared in their incorporation of spatial and phylogenetic autocorrelation, as well as their ability to account for assessor bias, including missing data and predicting specific IUCN categories. The method presented here is indicated as Caetano and colleagues [70]. IUCN, International Union for Conservation of Nature. (DOCX) [file pbio.3001544.s009.docx]

**S6 Table. Comparison of automated assessment methods.** Models are compared in their incorporation of spatial and phylogenetic autocorrelation, as well as their ability to account for assessor bias, including missing data and predicting specific IUCN categories. The method presented here is indicated as Caetano et al., 2022.

| Reference | Algorithm | Spatial Autocorrelation | Phylogenetic Autocorrelation | Assessor Bias | Allows Missing Data | Specific Categories |
| --- | --- | --- | --- | --- | --- | --- |
| Newton, 2010 | Bayesian Networks | no | no | no | yes | yes |
| Lee & Jetz, 2011 | Structural Equation Models | no | no | no | no | no |
| Bland et al, 2015 | Random Forest | no | no | no | no | no |
| Jetz & Freckleton, 2015 | Phylogenetic Regression | yes | yes | no | no | no |
| Pelletier et al, 2018 | Random Forest | no | no | no | no | no |
| González-del-Pliego et al, 2019 | Phylogenetic Regression | yes | yes | no | no | no |
| Miles, 2020 | Deep Learning | no | no | no | no | yes |
| Zizka et al, 2020 | Deep Learning | no | no | no | no | yes |
| Senior et al, 2021 | Bayesian Regression | no | yes | no | no | no |
| Caetano et al., 2022 | XGBoost | yes | yes | yes | yes | yes |
