# Supplementary material for: Automated assessment reveals that the extinction risk of reptiles is widely underestimated across space and phylogeny
Source: PLoS Biol. 2022 May 26;20(5):e3001544. doi: 10.1371/journal.pbio.3001544 (PMC9135251; doi:10.1371/journal.pbio.3001544)
Supplement: S7 Table — We adjusted p-values adjusted for false discovery rate. “Threatened” represents the proportion of species assigned a threatened category (CR, EN, and VU). Significant p-values are in bold. CR, Critically Endangered; DD, Data Deficient; EN, Endangered; LC, Least Concern; NE, Not Evaluated; NT, Near Threatened; VU, Vulnerable. (DOCX) [file pbio.3001544.s010.docx]

**S7 Table.** **Pearson’s Χ^2^ test statistics for comparisons of the proportion of reptile species assigned to each IUCN category between the actual assessments (Assessed) and the predictions for Data Deficient (DD) and Not Evaluated species (NE) species, made using an automated assessment model.** We adjusted p-values adjusted for False Discovery Rate. 'Threatened' represents the proportion of species assigned a threatened category (CR, EN and VU). CR – Critically Endangered, EN – Endangered, VU – Vulnerable, NT – Near Threatened, LC – Least Concern. Significant p-values are in bold.

| Category/ Comparison | χ^2^ | degrees of freedom | p-value | adjusted p-value |
| --- | --- | --- | --- | --- |
| Threatened | | | |  |
| Assessed vs. DD | 33.896 | 1 | < 0.001 | **< 0.001** |
| Assessed vs. NE | 26.777 | 1 | < 0.001 | **< 0.001** |
| DD vs. NE | 4.488 | 1 | 0.034 | 0.051 |
| CR |  |  |  |  |
| Assessed vs. DD | 8.218 | 1 | 0.004 | **0.008** |
| Assessed vs. NE | 1.353 | 1 | 0.245 | 0.283 |
| DD vs. NE | 3.362 | 1 | 0.067 | 0.092 |
| EN |  |  |  |  |
| Assessed vs. DD | 7.356 | 1 | 0.007 | **0.012** |
| Assessed vs. NE | 10.898 | 1 | < 0.001 | **0.002** |
| DD vs. NE | 0.180 | 1 | 0.672 | 0.672 |
| VU |  |  |  |  |
| Assessed vs. DD | 12.853 | 1 | < 0.001 | **0.002** |
| Assessed vs. NE | 11.243 | 1 | < 0.001 | **0.002** |
| DD vs. NE | 1.316 | 1 | 0.251 | 0.283 |
| NT |  |  |  |  |
| Assessed vs. DD | 11.911 | 1 | < 0.001 | **0.002** |
| Assessed vs. NE | 22.164 | 1 | < 0.001 | **< 0.001** |
| DD vs. NE | 0.244 | 1 | 0.621 | 0.658 |
| LC |  |  |  |  |
| Assessed vs. DD | 11.913 | 1 | < 0.001 | **0.002** |
| Assessed vs. NE | 5.276 | 1 | 0.022 | **0.035** |
| DD vs. NE | 3.139 | 1 | 0.076 | 0.098 |
