# Supplementary material for: Automated assessment reveals that the extinction risk of reptiles is widely underestimated across space and phylogeny
Source: PLoS Biol. 2022 May 26;20(5):e3001544. doi: 10.1371/journal.pbio.3001544 (PMC9135251; doi:10.1371/journal.pbio.3001544)
Supplement: S8 Table — IUCN, International Union for Conservation of Nature. (DOCX) [file pbio.3001544.s011.docx]

**S8 Table. Number of reptile species in each IUCN category before (rows) and after (columns) application of automated assessment method.**

|  | CR | EN | VU | NT | LC | **Total** |
| --- | --- | --- | --- | --- | --- | --- |
| CR | 209 | 55 | 27 | 2 | 10 | **303** |
| EN | 7 | 503 | 15 | 6 | 23 | **554** |
| VU | 7 | 21 | 450 | 2 | 38 | **518** |
| NT | 1 | 14 | 6 | 309 | 113 | **443** |
| LC | 4 | 15 | 9 | 33 | 4641 | **4702** |
| DD | 73 | 120 | 122 | 43 | 725 | **1083** |
| NE | 171 | 347 | 328 | 144 | 2296 | **3286** |
| **Total** | **472** | **1075** | **957** | **539** | **7846** | **10,889** |
