# Supplementary material for: Automated assessment reveals that the extinction risk of reptiles is widely underestimated across space and phylogeny
Source: PLoS Biol. 2022 May 26;20(5):e3001544. doi: 10.1371/journal.pbio.3001544 (PMC9135251; doi:10.1371/journal.pbio.3001544)
Supplement: S9 Table — DD, Data Deficient; NE, Not Evaluated. (DOCX) [file pbio.3001544.s012.docx]

**S9 Table. Difference in the proportion of threatened species in reptile families before and after the addition of extinction risk estimates for Data Deficient and Not Evaluated species, obtained from an automated assessment method.**

| Order | Family | Proportion before | Proportion after | Difference |
| --- | --- | --- | --- | --- |
| Crocodylia | Gavialidae | 1.000 | 1.000 | 0.000 |
| Crocodylia | Crocodylidae | 0.692 | 0.750 | 0.058 |
| Crocodylia | Alligatoridae | 0.125 | 0.125 | 0.000 |
| Testudina | Platysternidae | 1.000 | 1.000 | 0.000 |
| Testudina | Emydidae | 0.526 | 0.566 | 0.040 |
| Testudina | Testudinidae | 0.833 | 0.887 | 0.054 |
| Testudina | Geoemydidae | 0.800 | 0.803 | 0.003 |
| Testudina | Dermatemydidae | 1.000 | 1.000 | 0.000 |
| Testudina | Kinosternidae | 0.158 | 0.148 | -0.010 |
| Testudina | Chelydridae | 0.667 | 0.600 | -0.067 |
| Testudina | Carettochelyidae | 1.000 | 1.000 | 0.000 |
| Testudina | Trionychidae | 0.792 | 0.879 | 0.087 |
| Testudina | Chelidae | 0.500 | 0.293 | -0.207 |
| Testudina | Podocnemididae | 0.857 | 0.875 | 0.018 |
| Testudina | Pelomedusidae | 0.167 | 0.370 | 0.204 |
| Rhyncocephalia | Sphenodontidae | 0.000 | 0.000 | 0.000 |
| Squamata | Dibamidae | 0.222 | 0.320 | 0.098 |
| Squamata | Diplodactylidae | 0.244 | 0.258 | 0.014 |
| Squamata | Pygopodidae | 0.095 | 0.089 | -0.006 |
| Squamata | Carphodactylidae | 0.194 | 0.194 | 0.000 |
| Squamata | Eublepharidae | 0.480 | 0.395 | -0.085 |
| Squamata | Sphaerodactylidae | 0.328 | 0.320 | -0.008 |
| Squamata | Phyllodactylidae | 0.165 | 0.205 | 0.040 |
| Squamata | Gekkonidae | 0.221 | 0.237 | 0.016 |
| Squamata | Scincidae | 0.191 | 0.226 | 0.035 |
| Squamata | Cordylidae | 0.064 | 0.074 | 0.010 |
| Squamata | Gerrhosauridae | 0.269 | 0.216 | -0.053 |
| Squamata | Xantusiidae | 0.211 | 0.143 | -0.068 |
| Squamata | Teiidae | 0.104 | 0.111 | 0.007 |
| Squamata | Gymnophthalmidae | 0.199 | 0.219 | 0.020 |
| Squamata | Lacertidae | 0.201 | 0.176 | -0.025 |
| Squamata | Rhineuridae | 0.000 | 0.000 | 0.000 |
| Squamata | Bipedidae | 0.000 | 0.000 | 0.000 |
| Squamata | Cadeidae | 0.000 | 0.000 | 0.000 |
| Squamata | Amphisbaenidae | 0.222 | 0.237 | 0.015 |
| Squamata | Trogonophiidae | 0.000 | 0.000 | 0.000 |
| Squamata | Blanidae | 0.000 | 0.143 | 0.143 |
| Squamata | Varanidae | 0.130 | 0.139 | 0.009 |
| Squamata | Lanthanotidae | 0.000 | 0.000 | 0.000 |
| Squamata | Shinisauridae | 1.000 | 1.000 | 0.000 |
| Squamata | Xenosauridae | 0.600 | 0.583 | -0.017 |
| Squamata | Helodermatidae | 0.000 | 0.000 | 0.000 |
| Squamata | Anniellidae | 0.600 | 0.500 | -0.100 |
| Squamata | Diploglossidae | 0.436 | 0.391 | -0.045 |
| Squamata | Anguidae | 0.463 | 0.446 | -0.017 |
| Squamata | Chamaeleonidae | 0.387 | 0.404 | 0.016 |
| Squamata | Agamidae | 0.121 | 0.149 | 0.028 |
| Squamata | Phrynosomatidae | 0.131 | 0.139 | 0.008 |
| Squamata | Dactyloidae | 0.307 | 0.354 | 0.047 |
| Squamata | Corytophanidae | 0.000 | 0.182 | 0.182 |
| Squamata | Leiocephalidae | 0.333 | 0.419 | 0.086 |
| Squamata | Liolaemidae | 0.184 | 0.184 | 0.000 |
| Squamata | Leiosauridae | 0.214 | 0.176 | -0.038 |
| Squamata | Opluridae | 0.000 | 0.125 | 0.125 |
| Squamata | Tropiduridae | 0.137 | 0.161 | 0.023 |
| Squamata | Iguanidae | 0.738 | 0.705 | -0.034 |
| Squamata | Hoplocercidae | 0.333 | 0.316 | -0.018 |
| Squamata | Polychrotidae | 0.167 | 0.125 | -0.042 |
| Squamata | Crotaphytidae | 0.250 | 0.167 | -0.083 |
| Squamata | Leptotyphlopidae | 0.145 | 0.184 | 0.038 |
| Squamata | Gerrhopilidae | 0.000 | 0.421 | 0.421 |
| Squamata | Xenotyphlopidae | 1.000 | 0.000 | -1.000 |
| Squamata | Typhlopidae | 0.223 | 0.360 | 0.136 |
| Squamata | Anomalepididae | 0.000 | 0.150 | 0.150 |
| Squamata | Tropidophiidae | 0.556 | 0.545 | -0.010 |
| Squamata | Aniliidae | 0.000 | 0.000 | 0.000 |
| Squamata | Uropeltidae | 0.174 | 0.393 | 0.219 |
| Squamata | Anomochilidae | 0.000 | 0.333 | 0.333 |
| Squamata | Cylindrophiidae | 0.000 | 0.143 | 0.143 |
| Squamata | Xenopeltidae | 0.000 | 0.000 | 0.000 |
| Squamata | Pythonidae | 0.200 | 0.200 | 0.000 |
| Squamata | Loxocemidae | 0.000 | 0.000 | 0.000 |
| Squamata | Boidae | 0.281 | 0.210 | -0.072 |
| Squamata | Xenophidiidae | 0.000 | 0.000 | 0.000 |
| Squamata | Bolyeridae | 1.000 | 1.000 | 0.000 |
| Squamata | Acrochordidae | 0.000 | 0.000 | 0.000 |
| Squamata | Xenodermatidae | 0.143 | 0.318 | 0.175 |
| Squamata | Pareatidae | 0.000 | 0.100 | 0.100 |
| Squamata | Viperidae | 0.275 | 0.254 | -0.021 |
| Squamata | Homalopsidae | 0.130 | 0.302 | 0.171 |
| Squamata | Lamprophiidae | 0.158 | 0.131 | -0.026 |
| Squamata | Elapidae | 0.093 | 0.122 | 0.029 |
| Squamata | Colubridae | 0.098 | 0.151 | 0.053 |
| Squamata | Natricidae | 0.099 | 0.143 | 0.045 |
| Squamata | Pseudoxenodontidae | 0.000 | 0.000 | 0.000 |
| Squamata | Dipsadidae | 0.146 | 0.164 | 0.018 |
