# Supplementary material for: Automated assessment reveals that the extinction risk of reptiles is widely underestimated across space and phylogeny
Source: PLoS Biol. 2022 May 26;20(5):e3001544. doi: 10.1371/journal.pbio.3001544 (PMC9135251; doi:10.1371/journal.pbio.3001544)
Supplement: S10 Table — We adjusted p-values adjusted for false discovery rate. Significant p-values are in bold. DD, Data Deficient; NE, Not Evaluated. (DOCX) [file pbio.3001544.s013.docx]

**S10 Table. Pearson’s Χ^2^ test statistics for comparisons of the proportion of threatened reptile species in eight biogeographical realms, before and after the inclusion of predictions for Data Deficient and Not Evaluated species, made using an automated assessment model.** We adjusted p-values adjusted for False Discovery Rate. Significant p-values are in bold.

| Biogeographical Realm | Proportion before | Proportion after | Χ^2^ | degrees of freedom | p-value | adjusted p-value |
| --- | --- | --- | --- | --- | --- | --- |
| Australasian | 0.130 | 0.171 | 8.792 | 1 | 0.003 | **0.024** |
| Afrotropical | 0.146 | 0.136 | 0.270 | 1 | 0.603 | 0.769 |
| Indomalayan | 0.232 | 0.267 | 4.280 | 1 | 0.039 | 0.108 |
| Madagascan | 0.416 | 0.483 | 2.912 | 1 | 0.088 | 0.141 |
| Nearctic | 0.150 | 0.157 | 0.063 | 1 | 0.801 | 0.801 |
| Neotropical | 0.230 | 0.254 | 3.731 | 1 | 0.053 | 0.108 |
| Oceanian | 0.538 | 0.472 | 0.178 | 1 | 0.673 | 0.769 |
| Palearctic | 0.178 | 0.142 | 3.718 | 1 | 0.054 | 0.108 |
