# Supplementary material for: Automated assessment reveals that the extinction risk of reptiles is widely underestimated across space and phylogeny
Source: PLoS Biol. 2022 May 26;20(5):e3001544. doi: 10.1371/journal.pbio.3001544 (PMC9135251; doi:10.1371/journal.pbio.3001544)
Supplement: S11 Table — We adjusted p-values adjusted for false discovery rate. “Threatened” represents the proportion of species assigned a threatened category (CR, EN, and VU). Significant p-values are in bold. CR, Critically Endangered; EN, Endangered; LC, Least Concern; NT, Near Threatened; VU, Vulnerable. (DOCX) [file pbio.3001544.s014.docx]

**S11 Table. Pearson’s Χ^2^ test statistics for comparisons of the proportion of reptile species assigned to each IUCN category between the actual assessments (Observed) and the expected if the most optimist group of assessors assessed every species (Optimist) and if the most group pessimist assessed every species (Pessimist), estimated using an automated assessment model.** We adjusted p-values adjusted for False Discovery Rate. 'Threatened' represents the proportion of species assigned a threatened category (CR, EN and VU). CR – Critically Endangered, EN – Endangered, VU – Vulnerable, NT – Near Threatened, LC – Least Concern. Significant p-values are in bold.

| Category/Comparison | Χ^2^ | degrees of freedom | p-value | adjusted p-value |
| --- | --- | --- | --- | --- |
| Threatened |  |  |  |  |
| Observed vs. Optimist | 936.250 | 1 | < 0.001 | **< 0.001** |
| Observed vs. Pessimist | 307.500 | 1 | < 0.001 | **< 0.001** |
| Optimist vs. Pessimist | 2072.400 | 1 | < 0.001 | **< 0.001** |
| CR |  |  |  |  |
| Observed vs. Optimist | 218.780 | 1 | < 0.001 | **< 0.001** |
| Observed vs. Pessimist | 201.790 | 1 | < 0.001 | **< 0.001** |
| Optimist vs. Pessimist | 688.230 | 1 | < 0.001 | **< 0.001** |
| EN |  |  |  |  |
| Observed vs. Optimist | 415.400 | 1 | < 0.001 | **< 0.001** |
| Observed vs. Pessimist | 349.760 | 1 | < 0.001 | **< 0.001** |
| Optimist vs. Pessimist | 1261.400 | 1 | < 0.001 | **< 0.001** |
| VU |  |  |  |  |
| Observed vs. Optimist | 238.410 | 1 | < 0.001 | **< 0.001** |
| Observed vs. Pessimist | 117.780 | 1 | < 0.001 | **< 0.001** |
| Optimist vs. Pessimist | 25.643 | 1 | < 0.001 | **< 0.001** |
| NT |  |  |  |  |
| Observed vs. Optimist | 276.760 | 1 | < 0.001 | **< 0.001** |
| Observed vs. Pessimist | 114.360 | 1 | < 0.001 | **< 0.001** |
| Optimist vs. Pessimist | 652.830 | 1 | < 0.001 | **< 0.001** |
| LC |  |  |  |  |
| Observed vs. Optimist | 1299.400 | 1 | < 0.001 | **< 0.001** |
| Observed vs. Pessimist | 518.300 | 1 | < 0.001 | **< 0.001** |
| Optimist vs. Pessimist | 3083.900 | 1 | < 0.001 | **< 0.001** |
