# Supplementary material for: Automated assessment reveals that the extinction risk of reptiles is widely underestimated across space and phylogeny
Source: PLoS Biol. 2022 May 26;20(5):e3001544. doi: 10.1371/journal.pbio.3001544 (PMC9135251; doi:10.1371/journal.pbio.3001544)
Supplement: S12 Table — We adjusted p-values adjusted for false discovery rate. (DOCX) [file pbio.3001544.s015.docx]

**S12 Table. Pearson’s Χ^2^ test statistics for comparisons of the proportion of threatened reptile species in eight biogeographical realms between the actual assessments (Observed) and the expected if the most optimist group of assessors assessed every species (Optimist) and if the most group pessimist assessed every species (Pessimist), estimated using an automated assessment model.** We adjusted p-values adjusted for False Discovery Rate.

| Category/Comparison | Χ^2^ | degrees of freedom | p-value | adjusted p-value |
| --- | --- | --- | --- | --- |
| Australasian |  |  |  |  |
| Observed vs. Optimist | 86.411 | 1 | < 0.001 | **< 0.001** |
| Observed vs. Pessimist | 13.954 | 1 | < 0.001 | **< 0.001** |
| Optimist vs. Pessimist | 158.270 | 1 | < 0.001 | **< 0.001** |
| Afrotropical |  |  |  |  |
| Observed vs. Optimist | 28.319 | 1 | < 0.001 | **< 0.001** |
| Observed vs. Pessimist | 19.127 | 1 | < 0.001 | **< 0.001** |
| Optimist vs. Pessimist | 88.538 | 1 | < 0.001 | **< 0.001** |
| Indomalayan |  |  |  |  |
| Observed vs. Optimist | 114.530 | 1 | < 0.001 | **< 0.001** |
| Observed vs. Pessimist | 51.653 | 1 | < 0.001 | **< 0.001** |
| Optimist vs. Pessimist | 295.010 | 1 | < 0.001 | **< 0.001** |
| Madagascan |  |  |  |  |
| Observed vs. Optimist | 61.949 | 1 | < 0.001 | **< 0.001** |
| Observed vs. Pessimist | 3.769 | 1 | 0.052 | 0.063 |
| Optimist vs. Pessimist | 94.650 | 1 | < 0.001 | **< 0.001** |
| Nearctic |  |  |  |  |
| Observed vs. Optimist | 10.077 | 1 | < 0.001 | **0.002** |
| Observed vs. Pessimist | 3.423 | 1 | 0.064 | 0.073 |
| Optimist vs. Pessimist | 25.479 | 1 | < 0.001 | **< 0.001** |
| Neotropical |  |  |  |  |
| Observed vs. Optimist | 310.670 | 1 | < 0.001 | **< 0.001** |
| Observed vs. Pessimist | 122.320 | 1 | < 0.001 | **< 0.001** |
| Optimist vs. Pessimist | 734.300 | 1 | < 0.001 | **< 0.001** |
| Oceanian |  |  |  |  |
| Observed vs. Optimist | 1.866 | 1 | 0.172 | 0.179 |
| Observed vs. Pessimist | 0.000 | 1 | 1 | 1 |
| Optimist vs. Pessimist | 1.866 | 1 | 0.172 | 0.179 |
| Palearctic |  |  |  |  |
| Observed vs. Optimist | 41.993 | 1 | < 0.001 | **< 0.001** |
| Observed vs. Pessimist | 5.226 | 1 | 0.022 | **0.028** |
| Optimist vs. Pessimist | 74.736 | 1 | < 0.001 | **< 0.001** |
