# Supplementary material for: Automated assessment reveals that the extinction risk of reptiles is widely underestimated across space and phylogeny
Source: PLoS Biol. 2022 May 26;20(5):e3001544. doi: 10.1371/journal.pbio.3001544 (PMC9135251; doi:10.1371/journal.pbio.3001544)
Supplement: S1 Text — This includes strategies used for hyperparameter tuning and feature selection and model accuracies resulting from the use of a different optimization criteria for hyperparameter optimization and feature selection (F1-score instead of AUC) and a different classification algorithm (AdaBoost instead of XGBoost). (DOCX) [file pbio.3001544.s016.docx]

**Supporting Information for the paper: Automated assessment reveals that the extinction risk of reptiles is widely underestimated across space and phylogeny**

Caetano et al. 2022 PLoS Biology

**Hyperparameter tuning and feature selection**

A crucial step in XGBoost is hyperparameter tuning, which adjusts parameters regarding learning rates, tree architecture, subsampling, weighting and regularization [1, 2]. We tuned the hyperparameters learning rate (η), maximum tree depth, minimum child weight, row sampling, column sampling, weight balancing, and three regularization parameters (γ, α, λ) for each combination of range size class and classification task, using a heuristic approach. We started from default values (η: 0.3, maximum tree depth: 6, minimum child weight: 1, row sampling: 1, column sampling: 1, weight balancing: 1, γ: 0, α: 0 and λ: 1), then sequentially decreased or increased each parameter by 0.1 (within the bounds of possible values for each parameter; maximum tree depth was adjusted by 1, since it only admits integer values) and cross validated the resulting model, until the configuration providing the highest accuracy (measured by the area under the receiver operating characteristic curve - AUC) was reached. We repeated this procedure maximizing F-1 score instead of AUC, to assess if the choice of evaluation metric affected the results.

We then selected features to be used in the models by sequentially removing each feature and re-running the model for each combination of range size class and classification task, comparing the cross-validated AUC of each model and excluding the feature which elimination resulted in the greatest increase or no change in accuracy, then repeating this process until the removal of any of the remaining features caused loss in accuracy. We repeated this procedure maximizing F-1 score instead of AUC, to assess if the choice of evaluation affected the results.

**Alternative model results**

The alternative model using F1-score maximization instead of AUC had the same overall accuracy and F1-score (90% and 94%), but lower AUC (82%) for the binary task. Thus we proceeded with AUC maximization for the remaining models. The alternative model using the AdaBoost algorithm obtained lower predictive accuracy for the binary task (87%, AUC: 76%, F1-score: 92%), so we proceeded with the XGBoost algorithm.

**References**

1. Probst P, Bischl B, Boulesteix A-L. Tunability: Importance of hyperparameters of machine learning algorithms. arXiv Prepr arXiv180209596. 2018.
2. Chen T, Guestrin C. Xgboost: A scalable tree boosting system. Proceedings of the 22nd acm sigkdd international conference on knowledge discovery and data mining. 2016. pp. 785–794.
