# Supplementary material for: Automated assessment reveals that the extinction risk of reptiles is widely underestimated across space and phylogeny
Source: PLoS Biol. 2022 May 26;20(5):e3001544. doi: 10.1371/journal.pbio.3001544 (PMC9135251; doi:10.1371/journal.pbio.3001544)
Supplement: S2 Text — (PDF) [file pbio.3001544.s017.pdf]

Automated Assessment with XGBoost - tutorial

Gabriel Caetano, Uri Roll

2021-12-28

Contents

Set up **1**
Phylogenetic and spatial eigenvector maps 2

Training model **4**
Binary model 4
Specific categories 8

Predictions for DD and NE species **14**

In this tutorial, we will apply our automated assessment method (elaborated in the paper: Caetano et al. 2022 Automated assessment reveals extinction risk of reptiles is widely underestimated across space and phylogeny. PLoS Biology) to estimate IUCN threat category for the skinks of the world (Squamata, Scincidae).

Set up

Here are the packages that you need to install:

```
install.packages("sf")
install.packages("spdep")
install.packages("sp")
install.packages("parallel")
install.packages("pbapply")
install.packages("adespatial")
install.packages("MPSEM")
install.packages("missForest")
install.packages("caret")
install.packages("data.table")
install.packages("xgboost")
```

Load packages and data

```
library(magrittr)
```

We are using three main sources of data, which are readily available for most species: 1) A phylogeny of the Scincidae family (extracted from the Squamata phylogeny from Tonini et al, 2016). 2) Estimates of species distributions, obtained from the Global Assessment of Reptile Distributions (GARD). 3) Body mass and summaries of remotely sensed data (various publicly available sources) across each species distribution (GARD). See Supplemental Material of Caetano et al, 2022 for complete list of variables.

```
skink_tree <- readRDS("skink_tree.rds") # Phylogeny (Tonini et al, 2016)
skink_dist <- readRDS("skink_dist.rds") # Geographical distribution (GARD)
skink_data <- readRDS("skink_data.rds") # Species data (GARD, remotely sensed variables)
```

Phylogenetic and spatial eigenvector maps

We will use the distributions and phylogeny to calculate eigenvector maps, which represent the spatial and phylogenetic autocorrelation in the data.

Packages 'MPSEM' and 'adespatial' have excellent tutorials that explain the many nuances and options to calculate phylogenetic and spatial eigenvector maps:

- https://cran.r-project.org/web/packages/MPSEM/vignettes/MPSEM.pdf
- https://cran.r-project.org/web/packages/adespatial/vignettes/tutorial.html

First, we calculate the spatial eigenvector maps:

```r
# Create neighbourhood matrix
skink_nb <-
  skink_dist %>%
  sf::st_as_sf() %>%
  sf::st_geometry() %>%
  lapply(., function(x) sf::st_as_sfc(sf::st_bbox(x))[[1]]) %>%
  sf::st_as_sfc() %>%
  sf::st_intersects()

for (i in 1:length(skink_nb)) {
  if (length(skink_nb[[i]]) == 0L) skink_nb[[i]] <- 0L
  if (length(skink_nb[[i]]) > 1L) skink_nb[[i]] <- sort(skink_nb[[i]])
}

class(skink_nb) <- "nb"
attr(skink_nb, "region.id") <- skink_dist@data$Binomial
attr(skink_nb, "call") <- match.call()
attr(skink_nb, "type") <- "queen"
skink_nb <- spdep::sym.attr.nb(skink_nb)

# Weigh matrix by centroid distances
skink_nb_dist <- spdep::nbdists(skink_nb, sp::coordinates(skink_dist))

cl <- parallel::makeCluster(parallel::detectCores() - 1)
parallel::clusterExport(cl, c("skink_dist"))

dist_weights <- pbapply::pblapply(cl = cl, skink_nb_dist,
                                  function(x) 1 - x/max(dist(sp::coordinates(skink_dist))))

parallel::stopCluster(cl)

listw_skink <- spdep::nb2listw(skink_nb, glist = dist_weights, zero.policy = TRUE)

# Calculate Eigenvectors
MEM <- adespatial::mem(listw_skink)
```

Each eigenvector has an associated eigenvalue, which is proportional to the scale of the autocorrelation represented by the vector. To reduce the number of eigenvectors used in the model, we will select only those that represent broader scales. We will use only those with eigenvalue higher than 10% of the maximum eigenvalue.

```r
# Select only broader scale vectors (higher eigenvalues)
MEM_ev <- attr(MEM, "values")
```

```r
MEM_sel <- MEM[,MEM_ev > max(MEM_ev)*0.1]

# Reorder to match data table
MEM_sel <-
  MEM_sel[match(skink_data$binomial_gard_1_7, skink_dist$Binomial),]

# Save it so you won't have to do it again
saveRDS(MEM_sel, "MEM_skink.rds")
```

Next, we calculate the phyllogenetic eigenvector maps. We use a trait (body mass) to estimate some parameters in our evolutionary model. See 'MPSEM' vignette for more details on this approach.

```r
# Create phylogenetic tree graph

tree_graph <-
  MPSEM::Phylo2DirectedGraph(skink_tree)

# Reorder data so it is in the same order as the tree
skink_data_tree <-
  skink_data[skink_data$tonini_name %in% skink_tree$tip.label,]

# Get only species which are in the tree
skink_data_tree <-
  skink_data_tree[match(skink_tree$tip.label, skink_data_tree$tonini_name),]

# Here we use random forest to impute the missing body mass values,
# as the eigenvector function does not tolerate missing data
skink_data_tree <-
  missForest::missForest(skink_data_tree[-c(1, 171:174)])$ximp # remove non-numeric columns

# Calculate eigenvectors
PEM <-
  MPSEM::PEM.fitSimple(
    y = skink_data_tree$max_mass_g,
    x = NULL,
    w = tree_graph,
    d = "distance",
    sp = "species",
    lower = 0.1,
    upper = 1)

PEM_df <- as.data.frame(PEM)

names(PEM_df) <- paste0("PEM", 1:ncol(PEM_df))
```

We repeat the same selection based on eigenvalues.

```r
# Select only broader scale vectors (higher eigenvalues)
PEM_sel <- PEM_df[,PEM$d > max(PEM$d)*0.1]

# Reorder to match data table
PEM_sel <-
  PEM_sel[match(skink_data$tonini_name, skink_tree$tip.label),]

# Save it so you won't have to do it again
```

```
saveRDS(PEM_sel, "PEM_skink.rds")
```

Join all predictors into a single table:

```
pred_skink <-
  cbind(skink_data[-c(171:174)], # exclude taxonomy columns
        MEM_sel,
        PEM_sel)
```

Now we separate Not Evaluated and Data Deficient species from the remaining, which we are going to use to train our model. We also exclude extinct species.

```
pred_skink_df <-
  pred_skink[!skink_data$iucn_cat %in% c("NE", "DD", "EX", "EW"),]
pred_skink_ne_dd <-
  pred_skink[skink_data$iucn_cat %in% c("NE", "DD"),]
```

Training model

We use a hierarchical modeling framework, with the XGBoost algorithm separating species into specific threat categories. At each step, we perform hyperparameter tuning and feature selection. We also repeat the same procedure for four range size classes, based on IUCN B criterion (See Caetano et al, 2022 for more details).

Binary model

Our first step separates threatened from non-threatened species. There are several steps, including data processing, hyperparameter tuning and feature selection.

Data processing

First we separate data by range size class:

```
pred_skink_list <-
  list(
    gard_pred_big = pred_skink_df[pred_skink_df$range_size_km2 > 20000,],
    gard_pred_20k = pred_skink_df[pred_skink_df$range_size_km2 < 20000 &
                                  pred_skink_df$range_size_km2 > 5000,],
    gard_pred_5k = pred_skink_df[pred_skink_df$range_size_km2 < 5000 &
                                 pred_skink_df$range_size_km2 > 100,],
    gard_pred_100 = pred_skink_df[pred_skink_df$range_size_km2 < 100,]
  )
```

Before proceeding with the model, we must do some data processing, which involves partitioning the data into training and testing data sets, creating dummy variables for categorical features, and converting the data to XGBoost format. We will create a function for this, so we don't have to repeat it every time.

```
data_process <- function(x, cat){
    set.seed(1)

    # Partition training and testing data sets

    random_ind <- caret::createDataPartition(y = x[,cat], p = 0.8, list = FALSE)
    training_gard <- x[random_ind, ]
    testing_gard <- x[-random_ind, ]

    cat_tr <- training_gard[,cat]
```

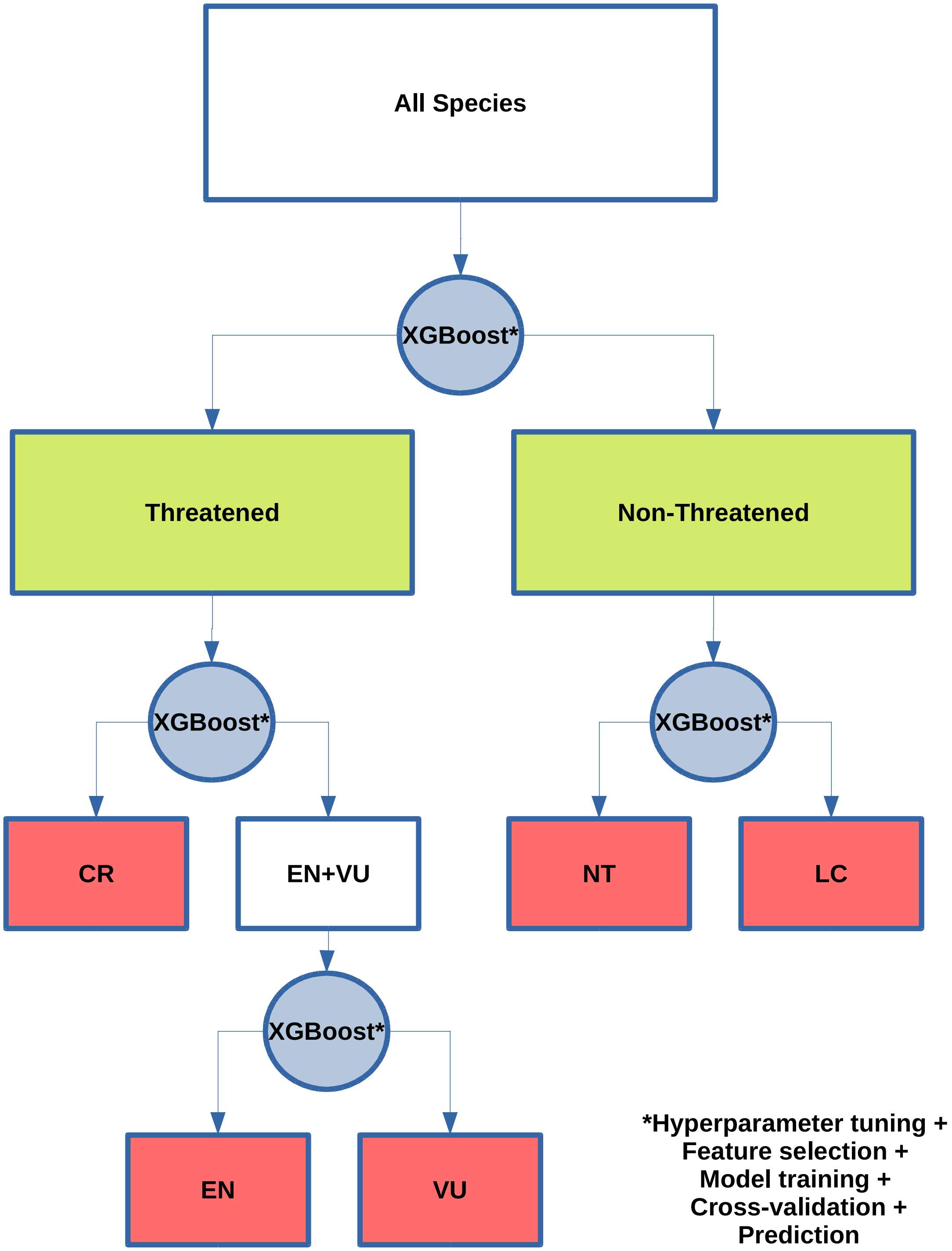

Figure 1: Modeling framework

```r
    cat_ts <- testing_gard[,cat]

    # create dummy variables for categorical features

    data.table::setDT(training_gard)
    data.table::setDT(testing_gard)

    tr_dummies <- caret::dummyVars(~., data = training_gard[,-c(cat),with=F])
    new_tr <- predict(tr_dummies, training_gard)

    ts_dummies <- caret::dummyVars(~., data = testing_gard[,-c(cat),with=F])
    new_ts <- predict(ts_dummies, testing_gard)

    # convert to XGBoost format

    which_cat <- which(names(training_gard) == cat)

    response_tr <- cat_tr %>% as.factor %>% {as.numeric(.) - 1}
    response_ts <- cat_ts %>% as.factor %>% {as.numeric(.) - 1}

    dtrain <- xgboost::xgb.DMatrix(data = new_tr, label = response_tr)
    dtest <- xgboost::xgb.DMatrix(data = new_ts, label = response_ts)

    list(dtrain = dtrain,
         dtest = dtest,
         response_tr = response_tr,
         response_ts = response_ts,
         new_tr = new_tr,
         new_ts = new_ts)
  }
```

```r
pred_skink_data <- lapply(pred_skink_list, data_process, cat = "threatened")
```

Hyperparameter tuning

Next we need to tune some hyperparameters in the model. These are modelling features such as learning rate, tree depth, weighting, sampling and regularization. See the XGBoost documentation for a description of parameters. We will create 10,000 random parameter samples and retain the one that leads to the highest AUC. There is a wealth of other hyperparameter tuning methods, we recommend researching into the alternatives so you can choose the most appropriate for your analysis.

```r
params_list <- lapply(pred_skink_data, hp_tuning)

saveRDS(params_list, "params_list_skink.rds")
```

```r
params_list <- readRDS("params_list_skink.rds")
```

Feature selection

There are many techniques for feature selection as well. Here we opted for a simple elimination heuristic, for the sake of simplicity:

```r
selected_cols <- feat_sel(data = pred_skink_data,
                          params = params_list)
```

```
saveRDS(selected_cols, "selected_cols_skink.rds")
```

```
selected_cols <- readRDS("selected_cols_skink.rds")
```

Now that we have the list of selected columns, we will subset them from the data:

```
pred_skink_data_sel <- pred_skink_data

for(i in 1:length(pred_skink_data_sel)){
pred_skink_data_sel[[i]]$new_tr <-
  pred_skink_data[[i]]$new_tr[,colnames(pred_skink_data[[i]]$new_tr) %in%
                              selected_cols[[i]]]
pred_skink_data_sel[[i]]$new_ts <-
  pred_skink_data[[i]]$new_ts[,colnames(pred_skink_data[[i]]$new_ts) %in%
                              selected_cols[[i]]]
}
```

Model fitting

Finally, we fit the XGBoost model:

```
xgb_list <-
  lapply(1:length(pred_skink_data_sel), function(i){

    dtrain <- xgboost::xgb.DMatrix(data = pred_skink_data_sel[[i]]$new_tr,
                                   label = pred_skink_data_sel[[i]]$response_tr)
    dtest <- xgboost::xgb.DMatrix(data = pred_skink_data_sel[[i]]$new_ts,
                                  label = pred_skink_data_sel[[i]]$response_ts)
    set.seed(1)

    xgb1 <-
      xgboost::xgb.train(params = params_list[[i]],
                         data = dtrain,
                         watchlist = list(val=dtest,
                                          train=dtrain),
                         print_every_n = 10,
                         early_stopping_rounds = 10,
                         maximize = F ,
                         eval_metric = "error",
                         nrounds=200)
  })
```

Cross validation

You can see in the output above that the model already gives us a sample cross validation, but we will perform an out of sample validation as well. First, we predict categories for the test data:

```
xgbpred_list  <-
  lapply(1:length(pred_skink_data_sel), function(i){

    dtest <- xgboost::xgb.DMatrix(data = pred_skink_data_sel[[i]]$new_ts,
                                  label = pred_skink_data_sel[[i]]$response_ts)

    #model prediction
    xgbpred <- predict (xgb_list[[i]], dtest)
    ifelse (xgbpred > 0.5,1,0)
```

```r
})
```

Then we calculate accuracy metrics:

```r
(confusion_mat <-
   caret::confusionMatrix(as.factor(unlist(xgbpred_list)),
                          as.factor(c(pred_skink_data_sel[[1]]$response_ts,
                                      pred_skink_data_sel[[2]]$response_ts,
                                      pred_skink_data_sel[[3]]$response_ts,
                                      pred_skink_data_sel[[4]]$response_ts)),
                          mode = "everything"))
```

```
## Confusion Matrix and Statistics
##
##           Reference
## Prediction   0   1
##          0 168  11
##          1   6  29
##
##                Accuracy : 0.9206
##                  95% CI : (0.8759, 0.953)
##     No Information Rate : 0.8131
##     P-Value [Acc > NIR] : 7.792e-06
##
##                   Kappa : 0.7254
##
##  Mcnemar's Test P-Value : 0.332
##
##             Sensitivity : 0.9655
##             Specificity : 0.7250
##          Pos Pred Value : 0.9385
##          Neg Pred Value : 0.8286
##               Precision : 0.9385
##                  Recall : 0.9655
##                      F1 : 0.9518
##              Prevalence : 0.8131
##          Detection Rate : 0.7850
##    Detection Prevalence : 0.8364
##       Balanced Accuracy : 0.8453
##
##        'Positive' Class : 0
##
```

```r
confusion_mat$byClass[11]
```

```
## Balanced Accuracy
##         0.8452586
```

We obtained a very high accuracy (92%) and high AUC (85%).

Specific categories

To train the model for specific categories, we repeat the steps above, but apply them to the subsets of data separated by the previous step, following the framework on Figure 1.

First we separate threatened from non-threatened species:

```
pred_skink_thrt <- pred_skink_df[pred_skink_df$threatened == "threatened",]
pred_skink_nthrt <- pred_skink_df[pred_skink_df$threatened == "non-threatened",]
```

CR vs EN+VU

We will then train the model to separate CR species from EN and VU, among the pool of threatened species. First we create a column indicating the CR species, to be our response variable

```
cr_ind <-
  skink_data[!is.na(skink_data$threatened) &
               skink_data$threatened == "threatened",]$iucn_cat == "CR"

pred_skink_thrt$cr <- 0
pred_skink_thrt$cr[cr_ind] <- 1

# remove the "threatened" column
pred_skink_thrt <- pred_skink_thrt[!names(pred_skink_thrt) %in% "threatened"]
```

Next we separate the data by range size classes. In this case, there are very insufficient CR species with range larger than 5,000 km^2 (only 2), so we will collapse the larger three range size classes.

```
pred_skink_list_thrt <-
  list(
    pred_skink_big = pred_skink_thrt[pred_skink_thrt$range_size_km2 > 100,],
    pred_skink_100 = pred_skink_thrt[pred_skink_thrt$range_size_km2 < 100,]
  )
```

We then follow the same steps as for the binary modelling. Data processing:

```
pred_skink_data_thrt <- lapply(pred_skink_list_thrt, data_process, "cr")
```

Hyperparameter tuning and feature selection:

```
params_list_cr <- lapply(pred_skink_data_thrt, hp_tuning)
selected_cols_cr <- feat_sel(pred_skink_data_thrt, params_list_cr)

saveRDS(params_list_cr, "params_list_cr.rds")
saveRDS(selected_cols_cr, "selected_cols_cr.rds")
```

```
params_list_cr <- readRDS("params_list_cr.rds")
selected_cols_cr <- readRDS("selected_cols_cr.rds")
```

Subset selected columns:

```
pred_skink_data_thrt_sel <- pred_skink_data_thrt

for(i in 1:length(pred_skink_data_thrt_sel)){
pred_skink_data_thrt_sel[[i]]$new_tr <-
  pred_skink_data_thrt_sel[[i]]$new_tr[,colnames(pred_skink_data_thrt_sel[[i]]$new_tr) %in%
                              selected_cols_cr[[i]]]
pred_skink_data_thrt_sel[[i]]$new_ts <-
  pred_skink_data_thrt_sel[[i]]$new_ts[,colnames(pred_skink_data_thrt_sel[[i]]$new_ts) %in%
                              selected_cols_cr[[i]]]
}
```

Train model:

```r
xgb_list_cr <-
  lapply(1:length(pred_skink_data_thrt_sel), function(i){

    dtrain <- xgboost::xgb.DMatrix(data = pred_skink_data_thrt_sel[[i]]$new_tr,
                                   label = pred_skink_data_thrt_sel[[i]]$response_tr)
    dtest <- xgboost::xgb.DMatrix(data = pred_skink_data_thrt_sel[[i]]$new_ts,
                                  label = pred_skink_data_thrt_sel[[i]]$response_ts)

    set.seed(1)

    xgb1 <-
      xgboost::xgb.train(params = params_list_cr[[i]],
                         data = dtrain,
                         watchlist = list(val=dtest,
                                          train=dtrain),
                         print_every_n = 10,
                         early_stopping_rounds = 10,
                         maximize = F ,
                         eval_metric = "error",
                         nrounds=200)
  })
```

Predict for testing set:

```r
xgbpred_list  <-
  lapply(1:length(pred_skink_data_thrt_sel), function(i){

    dtest <- xgboost::xgb.DMatrix(data = pred_skink_data_thrt_sel[[i]]$new_ts,
                                  label = pred_skink_data_thrt_sel[[i]]$response_ts)

    #model prediction
    xgbpred <- predict (xgb_list_cr[[i]], dtest)
    ifelse (xgbpred > 0.5,1,0)

  })
```

Cross validate:

```r
#confusion matrix
confusion_mat_cr <-
  caret::confusionMatrix(as.factor(unlist(xgbpred_list)),
                         as.factor(c(pred_skink_data_thrt_sel[[1]]$response_ts,
                                     pred_skink_data_thrt_sel[[2]]$response_ts)),
                         mode = "everything")

confusion_mat_cr$byClass[11]
```

```
## Balanced Accuracy
##         0.7354839
```

EN vs VU

We now repeat the same procedure, but for separating EN from VU species among the pool of threatened species left after removing CR species.

Create response variable:

```
en_ind <- skink_data[!is.na(skink_data$threatened) &
                       skink_data$threatened == "threatened",]$iucn_cat == "EN"

pred_skink_thrt$en <- 0
pred_skink_thrt$en[en_ind] <- 1

pred_skink_thrt_en <- pred_skink_thrt[pred_skink_thrt$cr == 0,]

pred_skink_thrt_en <-
  pred_skink_thrt_en[!names(pred_skink_thrt_en) %in% c("cr", "threatened")]
```

Separate range size classes:

```
pred_skink_list_thrt_en <-
  list(
    pred_skink_big = pred_skink_thrt_en[pred_skink_thrt_en$range_size_km2 > 20000,],
    pred_skink_20k = pred_skink_thrt_en[pred_skink_thrt_en$range_size_km2 < 20000 &
                                          pred_skink_thrt_en$range_size_km2 > 5000,],
    pred_skink_5k = pred_skink_thrt_en[pred_skink_thrt_en$range_size_km2 < 5000 &
                                         pred_skink_thrt_en$range_size_km2 > 100,],
    pred_skink_100 = pred_skink_thrt_en[pred_skink_thrt_en$range_size_km2 < 100,]
  )
```

Data processing:

```
pred_skink_data_thrt_en <- lapply(pred_skink_list_thrt_en, data_process, "en")
```

Hyperparameter tuning and feature selection:

```
params_list_en <- lapply(pred_skink_data_thrt_en, hp_tuning)
selected_cols_en <- feat_sel(pred_skink_data_thrt_en, params_list_en)

saveRDS(params_list_en, "params_list_en.rds")
saveRDS(selected_cols_en, "selected_cols_en.rds")
```

```
params_list_en <- readRDS("params_list_en.rds")
selected_cols_en <- readRDS("selected_cols_en.rds")
```

Subset selected columns:

```
pred_skink_data_thrt_en_sel <- pred_skink_data_thrt_en

for(i in 1:length(pred_skink_data_thrt_en_sel)){
pred_skink_data_thrt_en_sel[[i]]$new_tr <-
  pred_skink_data_thrt_en_sel[[i]]$new_tr[,colnames(pred_skink_data_thrt_en_sel[[i]]$new_tr) %in%
                               selected_cols_en[[i]]]
pred_skink_data_thrt_en_sel[[i]]$new_ts <-
  pred_skink_data_thrt_en_sel[[i]]$new_ts[,colnames(pred_skink_data_thrt_en_sel[[i]]$new_ts) %in%
                               selected_cols_en[[i]]]
}
```

Train model:

```
xgb_list_en <-
  lapply(1:length(pred_skink_data_thrt_en_sel), function(i){

    dtrain <- xgboost::xgb.DMatrix(data = pred_skink_data_thrt_en_sel[[i]]$new_tr,
                                   label = pred_skink_data_thrt_en_sel[[i]]$response_tr)
```

```
  dtest <- xgboost::xgb.DMatrix(data = pred_skink_data_thrt_en_sel[[i]]$new_ts,
                                label = pred_skink_data_thrt_en_sel[[i]]$response_ts)

  set.seed(1)

  xgb1 <-
    xgboost::xgb.train(params = params_list_en[[i]],
                       data = dtrain,
                       watchlist = list(val=dtest,
                                        train=dtrain),
                       print_every_n = 10,
                       early_stopping_rounds = 10,
                       maximize = F ,
                       eval_metric = "error",
                       nrounds=200)
})
```

Predict for testing set:

```
xgbpred_list  <-
  lapply(1:length(pred_skink_data_thrt_en_sel), function(i){

    dtest <- xgboost::xgb.DMatrix(data = pred_skink_data_thrt_en_sel[[i]]$new_ts,
                                  label = pred_skink_data_thrt_en_sel[[i]]$response_ts)

    #model prediction
    xgbpred <- predict (xgb_list_en[[i]], dtest)
    ifelse (xgbpred > 0.5,1,0)

  })
```

Cross-validate:

```
#confusion matrix
confusion_mat_en <-
  caret::confusionMatrix(as.factor(unlist(xgbpred_list)),
                         as.factor(c(pred_skink_data_thrt_en_sel[[1]]$response_ts,
                                     pred_skink_data_thrt_en_sel[[2]]$response_ts,
                                     pred_skink_data_thrt_en_sel[[3]]$response_ts,
                                     pred_skink_data_thrt_en_sel[[4]]$response_ts)))

confusion_mat_en$byClass[11]
```

```
## Balanced Accuracy
##         0.8990385
```

NT vs LC

Finally, we train the model to separate the two non-threatened categories, NT and LC. It follows the same steps as above:

Create response variable:

```
nt_ind <- skink_data[!is.na(skink_data$threatened) &
                       skink_data$threatened == "non-threatened",]$iucn_cat == "NT"

pred_skink_nthrt$nt <- 0
```

```
pred_skink_nthrt$nt[nt_ind] <- 1

pred_skink_nthrt <- pred_skink_nthrt[!names(pred_skink_nthrt) %in% "threatened"]
```

Separate by range size class:

```
pred_skink_list_nthrt <-
  list(
    pred_skink_big = pred_skink_nthrt[pred_skink_nthrt$range_size_km2 > 20000,],
    pred_skink_20k = pred_skink_nthrt[pred_skink_nthrt$range_size_km2 < 20000 &
                                       pred_skink_nthrt$range_size_km2 > 5000,],
    pred_skink_5k = pred_skink_nthrt[pred_skink_nthrt$range_size_km2 < 5000 &
                                      pred_skink_nthrt$range_size_km2 > 100,],
    pred_skink_100 = pred_skink_nthrt[pred_skink_nthrt$range_size_km2 < 100,]
  )
```

Data processing:

```
pred_skink_data_nthrt <- lapply(pred_skink_list_nthrt, data_process, "nt")
```

Hyperparameter tuning and feature selection:

```
params_list_nt <- lapply(pred_skink_data_nthrt, hp_tuning)
selected_cols_nt <- feat_sel(pred_skink_data_nthrt, params_list_nt)

saveRDS(params_list_nt, "params_list_nt.rds")
saveRDS(selected_cols_nt, "selected_cols_nt.rds")
```

```
params_list_nt <- readRDS("params_list_nt.rds")
selected_cols_nt <- readRDS("selected_cols_nt.rds")
```

Subset selected columns:

```
pred_skink_data_nthrt_sel <- pred_skink_data_nthrt

for(i in 1:length(pred_skink_data_nthrt_sel)){
pred_skink_data_nthrt_sel[[i]]$new_tr <-
  pred_skink_data_nthrt_sel[[i]]$new_tr[,colnames(pred_skink_data_nthrt_sel[[i]]$new_tr) %in%
                                 selected_cols_nt[[i]]]
pred_skink_data_nthrt_sel[[i]]$new_ts <-
  pred_skink_data_nthrt_sel[[i]]$new_ts[,colnames(pred_skink_data_nthrt_sel[[i]]$new_ts) %in%
                                 selected_cols_nt[[i]]]
}
```

Train model:

```
xgb_list_nt <-
  lapply(1:length(pred_skink_data_nthrt_sel), function(i){

    dtrain <- xgboost::xgb.DMatrix(data = pred_skink_data_nthrt_sel[[i]]$new_tr,
                                   label = pred_skink_data_nthrt_sel[[i]]$response_tr)
    dtest <- xgboost::xgb.DMatrix(data = pred_skink_data_nthrt_sel[[i]]$new_ts,
                                  label = pred_skink_data_nthrt_sel[[i]]$response_ts)

    set.seed(1)

    xgb1 <-
      xgboost::xgb.train(params = params_list_nt[[i]],
```

```
                          data = dtrain,
                          watchlist = list(val=dtest,
                                           train=dtrain),
                          print_every_n = 10,
                          early_stopping_rounds = 10,
                          maximize = F ,
                          eval_metric = "error",
                          nrounds=200)
  })
```

Predict for testing set:

```
xgbpred_list  <-
  lapply(1:length(pred_skink_data_nthrt_sel), function(i){

    dtest <- xgboost::xgb.DMatrix(data = pred_skink_data_nthrt_sel[[i]]$new_ts,
                                   label = pred_skink_data_nthrt_sel[[i]]$response_ts)

    #model prediction
    xgbpred <- predict (xgb_list_nt[[i]], dtest)
    ifelse (xgbpred > 0.5,1,0)

  })
```

Cross-validate:

```
#confusion matrix
confusion_mat_nt <-
  caret::confusionMatrix(as.factor(unlist(xgbpred_list)),
                         as.factor(c(pred_skink_data_nthrt_sel[[1]]$response_ts,
                                     pred_skink_data_nthrt_sel[[2]]$response_ts,
                                     pred_skink_data_nthrt_sel[[3]]$response_ts,
                                     pred_skink_data_nthrt_sel[[4]]$response_ts)))

confusion_mat_nt$byClass[11]
```

```
## Balanced Accuracy
##         0.8469512
```

Predictions for DD and NE species

Now that we finished training our model, we can use it to predict the threat categories of DD and NE species. First, we must add an identification column, so we can later associate predictions with species.

```
pred_skink_ne_dd$id <-
  skink_data$gard_1_7_taxon_id[skink_data$iucn_cat %in% c("NE", "DD")]
```

Next, we separate them by range size class:

```
pred_skink_list_ne <-
  list(
    pred_skink_big = pred_skink_ne_dd[pred_skink_ne_dd$range_size_km2 > 20000,],
    pred_skink_20k = pred_skink_ne_dd[pred_skink_ne_dd$range_size_km2 < 20000 &
                                       pred_skink_ne_dd$range_size_km2 > 5000,],
    pred_skink_5k = pred_skink_ne_dd[pred_skink_ne_dd$range_size_km2 < 5000 &
                                      pred_skink_ne_dd$range_size_km2 > 100,],
```

```
    pred_skink_100 = pred_skink_ne_dd[pred_skink_ne_dd$range_size_km2 < 100,]
  )
```

Data processing:

```
gard_pred_data_ne <-
  lapply(pred_skink_list_ne, function(x){
    set.seed(1)

    gard_NE <- x
    data.table::setDT(gard_NE)

    library(caret)
    ne_dummies <- caret::dummyVars(~., data = gard_NE[,-c("threatened", "id"),with=F])
    new_ne <- predict(ne_dummies, gard_NE)

    list(id = gard_NE$id,
         new_ne = new_ne)
  })
```

```
## Loading required package: ggplot2
## Loading required package: lattice
```

Then we apply our binary model to obtain the threatened/non-threatened predictions:

```
xgbpred_list_ne  <-
  lapply(1:4, function(i){

    data_ne <- gard_pred_data_ne[[i]]$new_ne
    data_ne <- data_ne[,colnames(data_ne) %in% xgb_list[[i]]$feature_names]

    dne <- xgboost::xgb.DMatrix(data = data_ne)

    #model prediction
    xgbpred <- predict(xgb_list[[i]], dne)
    ifelse (xgbpred > 0.5,1,0)

  })
```

We separate those predicted to be threatened from those predicted to be non-threatened:

```
gard_pred_data_thrt_ne <-
  lapply(1:length(xgbpred_list_ne), function(i){

    new_ne <- gard_pred_data_ne[[i]]$new_ne[xgbpred_list_ne[[i]] == 1,]
    id <- gard_pred_data_ne[[i]]$id[xgbpred_list_ne[[i]] == 1]

    list(id = id,
         new_ne = new_ne)
  })

gard_pred_data_nthrt_ne <-
  lapply(1:length(xgbpred_list_ne), function(i){

    new_ne <- gard_pred_data_ne[[i]]$new_ne[xgbpred_list_ne[[i]] == 0,]
    id <- gard_pred_data_ne[[i]]$id[xgbpred_list_ne[[i]] == 0]
```

```
    list(id = id,
         new_ne = new_ne)
  })
```

Now from the pool of threatened species, we predict which are CR. Note that the model for CR had only two range size classes, due to insufficient sampling, so we apply the same model for the three range size classes above 100 km^2.

```
xgb_list_cr <- xgb_list_cr[c(1, 1, 1, 2)] # repeat first model three times

xgbpred_list_cr_ne  <-
  lapply(1:4, function(i){

    if(all(xgbpred_list_ne[[i]] == 0)) return(NULL)

    data_ne <- gard_pred_data_ne[[i]]$new_ne[xgbpred_list_ne[[i]] == 1,]
    data_ne <- data_ne[,colnames(data_ne) %in% xgb_list_cr[[i]]$feature_names]

    dtest <- xgboost::xgb.DMatrix(data = data_ne)

    #model prediction
    xgbpred <- predict(xgb_list_cr[[i]], dtest)
    ifelse (xgbpred > 0.5,1,0)

  })
```

Now we separate those predicted to be CR from the remaining threatened species:

```
gard_pred_data_cr_ne <-
  lapply(1:length(xgbpred_list_cr_ne), function(i){

    new_ne <- gard_pred_data_thrt_ne[[i]]$new_ne[xgbpred_list_cr_ne[[i]] == 1,]
    id <- gard_pred_data_thrt_ne[[i]]$id[xgbpred_list_cr_ne[[i]] == 1]

    list(id = id,
         new_ne = new_ne)
  })

gard_pred_data_ncr_ne <-
  lapply(1:length(xgbpred_list_cr_ne), function(i){

    new_ne <- gard_pred_data_thrt_ne[[i]]$new_ne[xgbpred_list_cr_ne[[i]] == 0,]
    id <- gard_pred_data_thrt_ne[[i]]$id[xgbpred_list_cr_ne[[i]] == 0]

    list(id = id,
         new_ne = new_ne)
  })
```

And apply the EN vs VU model to predict the categories of the remaining species:

```
xgbpred_list_en_ne  <-
  lapply(1:4, function(i){

    if(all(xgbpred_list_cr_ne[[i]] == 1)) return(NULL)

    data_ne <- gard_pred_data_thrt_ne[[i]]$new_ne[xgbpred_list_cr_ne[[i]] == 0,]
```

```
    data_ne <- data_ne[,colnames(data_ne) %in% xgb_list_en[[i]]$feature_names]

    dtest <- xgboost::xgb.DMatrix(data = data_ne)

    #model prediction
    xgbpred <- predict(xgb_list_en[[i]], dtest)
    ifelse (xgbpred > 0.5,1,0)

  })
```

Separate those predicted to be EN from those predicted to be VU:

```
gard_pred_data_en_ne <-
  lapply(1:length(xgbpred_list_cr_ne), function(i){

    new_ne <- gard_pred_data_ncr_ne[[i]]$new_ne[xgbpred_list_en_ne[[i]] == 1,]
    id <- gard_pred_data_ncr_ne[[i]]$id[xgbpred_list_en_ne[[i]] == 1]

    list(id = id,
         new_ne = new_ne)
  })

gard_pred_data_vu_ne <-
  lapply(1:length(xgbpred_list_cr_ne), function(i){

    new_ne <- gard_pred_data_ncr_ne[[i]]$new_ne[xgbpred_list_en_ne[[i]] == 0,]
    id <- gard_pred_data_ncr_ne[[i]]$id[xgbpred_list_en_ne[[i]] == 0]

    list(id = id,
         new_ne = new_ne)
  })
```

Finally, use the last model to predict which of the non-threatened species are NT and which are LC:

```
xgbpred_list_nt_ne  <-
  lapply(1:4, function(i){

    data_ne <- gard_pred_data_ne[[i]]$new_ne[xgbpred_list_ne[[i]] == 0,]
    data_ne <- data_ne[,colnames(data_ne) %in% xgb_list_nt[[i]]$feature_names]

    dtest <- xgboost::xgb.DMatrix(data = data_ne)

    #model prediction
    xgbpred <- predict(xgb_list_nt[[i]], dtest)
    ifelse (xgbpred > 0.5,1,0)

  })
```

Separate NT from LC predictions:

```
gard_pred_data_nt_ne <-
  lapply(1:length(xgbpred_list_nt_ne), function(i){

    new_ne <- gard_pred_data_nthrt_ne[[i]]$new_ne[xgbpred_list_nt_ne[[i]] == 1,]
    id <- gard_pred_data_nthrt_ne[[i]]$id[xgbpred_list_nt_ne[[i]] == 1]
```

```
    list(id = id,
         new_ne = new_ne)
  })

gard_pred_data_lc_ne <-
  lapply(1:length(xgbpred_list_nt_ne), function(i){

    new_ne <- gard_pred_data_nthrt_ne[[i]]$new_ne[xgbpred_list_nt_ne[[i]] == 0,]
    id <- gard_pred_data_nthrt_ne[[i]]$id[xgbpred_list_nt_ne[[i]] == 0]

    list(id = id,
         new_ne = new_ne)
  })
```

Now we group all predictions into a single table:

```
pred_id_cr_ne <- gard_pred_data_cr_ne %>% lapply(`[[`, 1) %>% unlist
pred_id_en_ne <- gard_pred_data_en_ne %>% lapply(`[[`, 1) %>% unlist
pred_id_vu_ne <- gard_pred_data_vu_ne %>% lapply(`[[`, 1) %>% unlist
pred_id_nt_ne <- gard_pred_data_nt_ne %>% lapply(`[[`, 1) %>% unlist
pred_id_lc_ne <- gard_pred_data_lc_ne %>% lapply(`[[`, 1) %>% unlist

pred_table <-
  rbind(
cbind(pred_id_cr_ne,
      skink_data$iucn_cat[match(pred_id_cr_ne,
                                skink_data$gard_1_7_taxon_id)], "CR"),
cbind(pred_id_en_ne,
      skink_data$iucn_cat[match(pred_id_en_ne,
                                skink_data$gard_1_7_taxon_id)], "EN"),
cbind(pred_id_vu_ne,
      skink_data$iucn_cat[match(pred_id_vu_ne,
                                skink_data$gard_1_7_taxon_id)], "VU"),
cbind(pred_id_nt_ne,
      skink_data$iucn_cat[match(pred_id_nt_ne,
                                skink_data$gard_1_7_taxon_id)], "NT"),
cbind(pred_id_lc_ne,
      skink_data$iucn_cat[match(pred_id_lc_ne,
                                skink_data$gard_1_7_taxon_id)], "LC")
) %>% as.data.frame()

names(pred_table) <- c("gard_1_7_id", "original_cat", "predicted_cat")

pred_table$binomial <-
  skink_data$binomial_gard_1_7[match(pred_table$gard_1_7_id,
                              skink_data$gard_1_7_taxon_id)]

head(pred_table)
```

```
##   gard_1_7_id original_cat predicted_cat                   binomial
## 1      R11412            6            CR       Acontias albigularis
## 2      R11439            7            CR        Acontias parietalis
## 3      R07075            7            CR   Asymblepharus nepalensis
## 4      R07077            7            CR Asymblepharus tragbulense
```

```
## 5      R10386            7            CR Brachymeles dalawangdaliri
## 6      R07100            7            CR    Brachymeles isangdaliri
```

We can plot the results in bar charts to examine the distribution of categories in the original data set and in our predictions for DD and NE species.

```
# Original categories
skink_data$iucn_cat <-
  factor(skink_data$iucn_cat,
         levels = c("CR", "EN", "VU", "NT", "LC", "DD", "NE", "EX", "EW"))

ggplot2::ggplot(skink_data) +
  ggplot2::geom_bar(aes(x = iucn_cat)) +
  ggplot2::theme_classic()
```

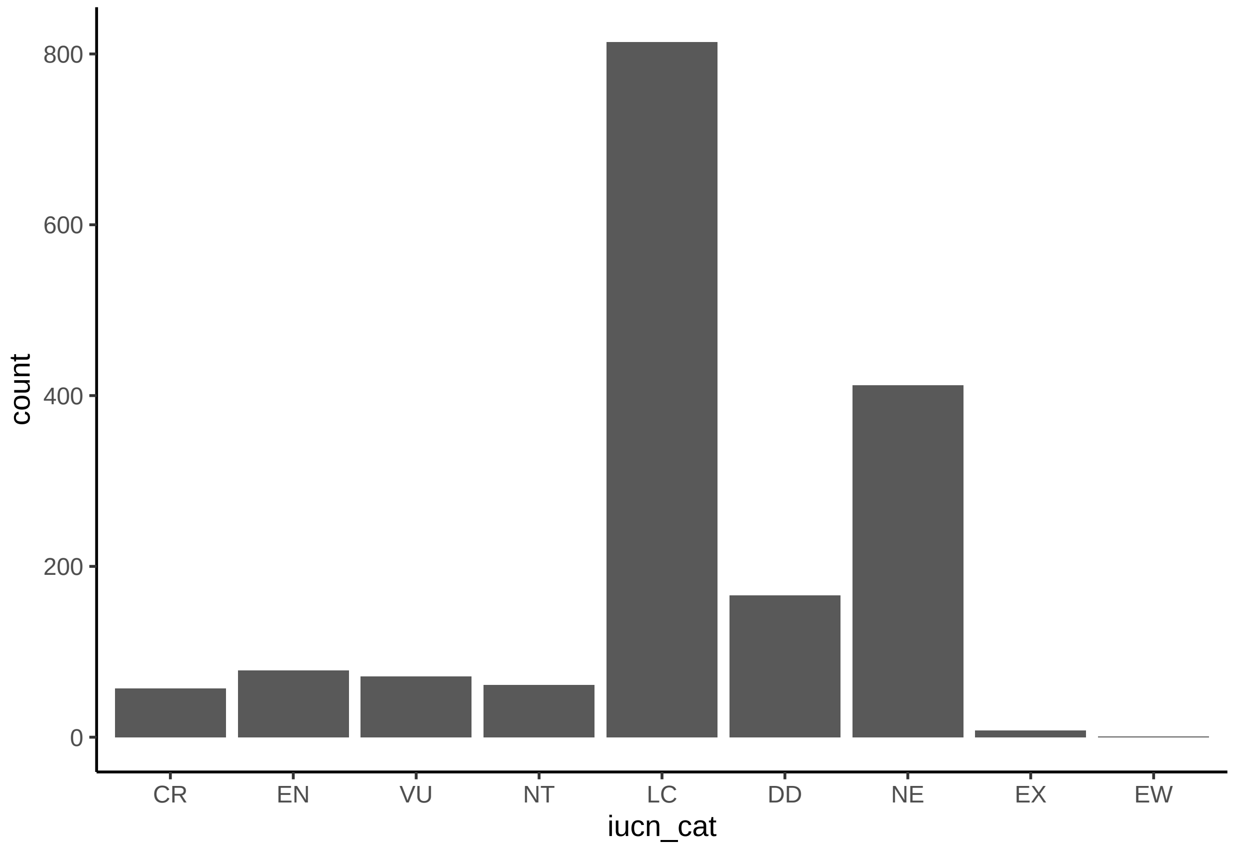


```
# Predictions for DD and NE species
pred_table$predicted_cat <-
  factor(pred_table$predicted_cat,
         levels = c("CR", "EN", "VU", "NT", "LC"))

ggplot2::ggplot(pred_table) +
  ggplot2::geom_bar(aes(x = predicted_cat)) +
  ggplot2::theme_classic()
```

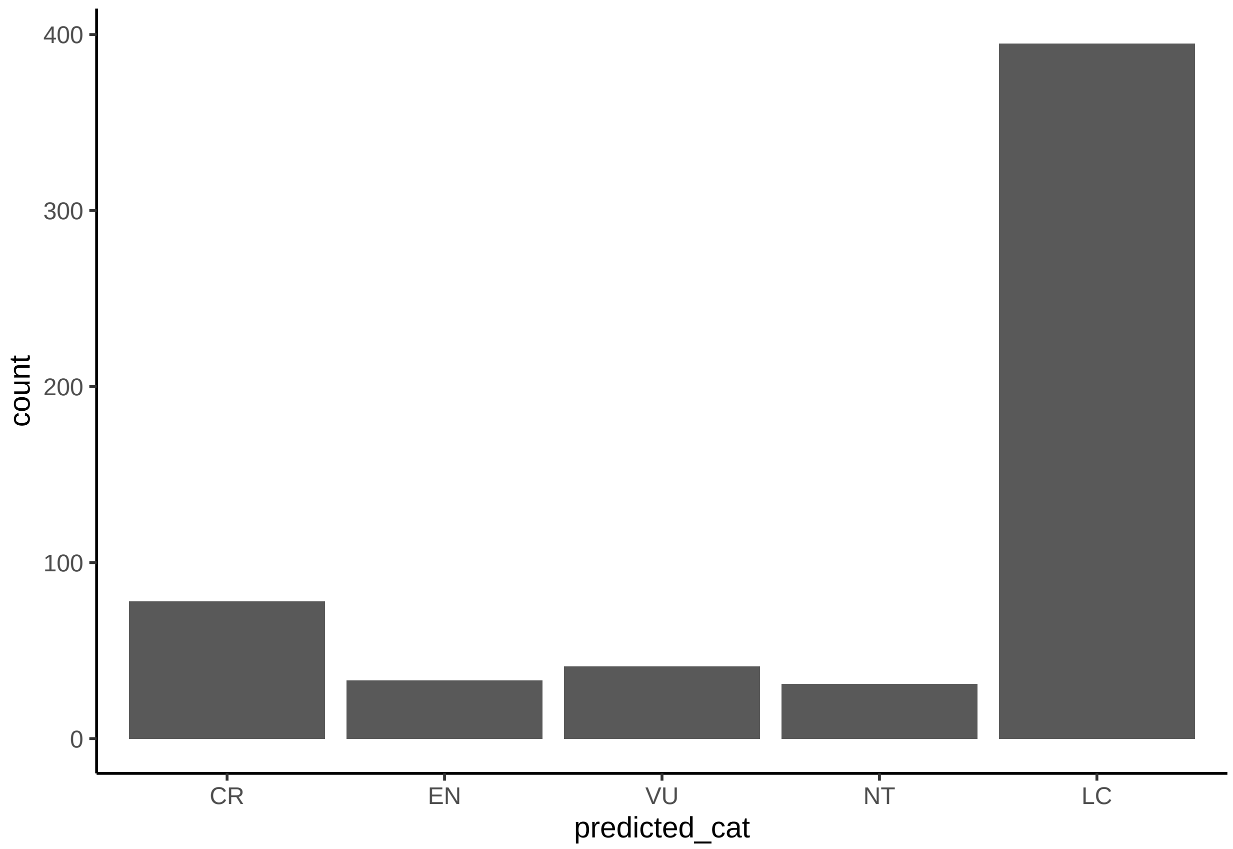
count
400
300
200
100
0
CR
EN
VU
NT
LC
predicted_cat
